# Exploring global research status and trends in anti-obesity effects of traditional Chinese medicine through intestinal microbiota: a bibliometric study

**DOI:** 10.3389/fcimb.2023.1271473

**Published:** 2023-11-16

**Authors:** Wenjing Huang, Jiuyuan Wang, Min Kuang, Zixuan Xiao, Boyan Fan, Guixiang Sun, Zhoujin Tan

**Affiliations:** College of Chinese Medicine, Hunan University of Chinese Medicine, Changsha, China

**Keywords:** traditional Chinese medicine, intestinal microbiota, obesity, bibliometric analysis, VOSviewer, CiteSpace, research status and trends

## Abstract

**Background:**

The intestinal microbiota (IM) has been found to contribute to metabolic disorders that lead to excessive fat accumulation, systemic and chronic low-grade inflammation, and insulin resistance in the host. Current research highlights a pivotal interaction between IM and traditional Chinese medicine (TCM) in mitigating obesity-related diseases. Undeniably, IM stands as a central focus in TCM research aimed at preventing and treating obesity. Therefore, tracing the progress and trends in this field can offer valuable references and insights for future studies.

**Methods:**

On June 17, 2023, we conducted a literature search on the topic of “IM and obesity in TCM” spanning the period from 2009 to 2023. We extracted the primary information of the publications, which includes complete records and reference citations, from the Science Citation Index Expanded (SCI-E) within the Web of Science Core Collection (WoSCC). To visualize and analyze the literature, we utilized CiteSpace and VOSviewer for bibliometric analysis.

**Results:**

During the past fifteen years, a rapid increase in the number of publications has been observed. The cooperative networks demonstrate China, Beijing University of Chinese Medicine, and Food & Function as the most active countries, organizations, and journals in this field, respectively. Liu Bin has contributed the most publications. A paper by Xu Jia, published in 2014, holds the highest Local Citation Score (LCS). Analyses of keyword co-occurrence and reference co-citation indicate that the research hotspots of IM and obesity in TCM are primarily focused on the metabolic benefits driven by endogenous functional metabolic molecules generated by TCM regulation of IM. Other focal points include the mechanism by which TCM regulates IM to restore the intestinal mucosal barrier This is a provisional file, not the final typeset article, and manages the gut-organ axis, the metabolic advantages of acupuncture’s regulation of IM, and the process by which Chinese medicine small molecules transform IM.

**Conclusion:**

This research offers a comprehensive understanding of the current status, hotspots, and trends in global TCM research. Additionally, it provides a comprehensive summary and exploration of the latest advancements in this field, thereby emphasizing the essence of TCM more effectively.

## 1 Introduction

Obesity has escalated into a global public health crisis with the worldwide prevalence doubling since 1975 (Lim et al., 2012). Particularly in China, there has been a rapid surge in overweight and obesity rates, and the associated chronic diseases over the past two decades (Wang et al., 2021). Obesity elevates the risk for numerous health complications such as type 2 diabetes (T2D), cardiovascular diseases, dyslipidemia, sleep apnea, mental health disorders, and a variety of cancers including breast, ovarian, renal, and hepatic cancers (Gallagher and LeRoith, 2015; Lauby-Secretan et al., 2016; Afshin et al., 2017). As our understanding of the mechanisms underlying obesity deepens, the treatment approaches have advanced rapidly. Managing obesity, like all chronic diseases, demands sustained multi-modal strategies. Recognizing the unique treatment objectives of individuals, diverse therapeutic methods present distinct benefits and potential risks (Perdomo et al., 2023).

Recent studies have substantiated a strong correlation between IM and obesity onset (Zhao, 2013). Often considered the ninth largest organ system and the second largest genome in the human body, IM’s bacterial content surpasses the total cell count, earning it recognition as another “organ”. In obesity, a shift in the IM composition manifests with a decrease in Bacteroidetes and an increase in Firmicutes. This alteration correlates with body weight and fat accumulation, suggesting a micro-ecological imbalance in the gut of most obese patients (Nerurkar et al., 2019). Additionally, imbalances in gut microbes trigger a series of obesity-related inflammatory responses, oxidative stress, and metabolic dysfunctions within the host (Rajani and Jia, 2018).

TCM offers thousands of years of clinical experience in managing obesity, with the benefit of multi-pathway, multi-target, and holistic regulation (Wei, 1992; Guo et al., 2002; Zhang CH. et al., 2021). Meanwhile, TCM can intervene in numerous diseases, including obesity, by regulating the proportion and structure of the IM (Yue et al., 2019; Zhang et al., 2020; Li D. et al., 2023). The research domain on IM and obesity in TCM has garnered significant interest. Given that most TCM remedies are ingested orally, the active ingredients interact with IM upon entering the gastrointestinal tract. Some studies have systematically reviewed and analyzed these interactions, providing a comprehensive understanding of TCM’s role in IM regulation and its implications (Li et al., 2021; Su et al., 2021). They explore TCM’s potential mechanism in correcting metabolic disorders by adjusting the composition and functional structure of IM, especially pertaining to intestinal barrier function and metabolites (Zhang HY. et al., 2021). Furthermore, research has summarized the molecular mechanisms of TCM regulation of IM in obesity treatment (Zhang et al., 2020). While these contributions are substantial, there exist certain limitations in content and methodology, such as the lack of comparative analysis and over-reliance on subjective interpretations.

Addressing these limitations, thoroughly understanding, comparison, and analysis of research hotspots and development trajectories in this field can significantly bolster theoretical research and practical innovation in TCM. As far as we know, no bibliometric studies have been reported to assess the hotspots and research trends of TCM intervention in IM for obesity prevention and treatment. Therefore, this research leverages acclaimed bibliometric software like VOSviewer and CiteSpace for analysis (Yang et al., 2022; Zhang et al., 2022; Wei et al., 2023). In the course of our investigation, we observed a consistent increase in the quantity of studies pertaining to TCM intervention in IM for obesity prevention and treatment since 2009. Consequently, we executed a visual comparative analysis of the research outcomes spanning 2009 to 2023. The aim was to objectively delineate the variations and commonalities in the research areas of different teams, thereby providing valuable insights and references for future endeavors in TCM intervention in IM for obesity prevention and treatment.

## 2 Materials and methods

### 2.1 Data source and methodology

The WoS encompasses a broad array of publications from various fields, acknowledged and utilized by numerous researchers as the most appropriate database for bibliometric analysis (Ding and Yang, 2020).Therefore, this study selected the WoSCC as the source of data. To ensure the breadth and accuracy of data collection, the SCI-E was implemented. The period covered is from January 2009 to June 2023, and the time up until search was conducted was June 17, 2023. All searches were executed on the same day to avoid any major discrepancies due to database upgrades. We optimized our search results using keywords, including “intestinal microbiota”, “obesity”, and “TCM”, combined with Boolean operators (“AND”, “OR”). The search formula we used is as follows: (((TS=(gut OR bowel OR colon OR intestine* OR gastrointestin* OR gastro-intestine*)) AND TS=(micro bio* OR microbiome* OR microbiota OR microflora OR flora OR bacteria* OR commensal OR probiotic OR prebiotic OR microecology OR dysbiosis OR pathobiont)) AND TS=(obesity OR high-fat diet OR over weight OR overweight OR obese OR body mass index OR BMI)) AND TS=(Chinese medicine OR traditional Chinese medicine OR TCM OR herbal medicine OR material medicine OR decoction OR powder OR pill OR extract OR Chinese proprietary medicine). From the search results, we initially excluded studies that failed to meet the criteria based on title and abstract review. We then ensured the inclusion criteria were satisfied through full-text reading. This process was undertaken by two independent reviewers, and in case of any disagreement, a third reviewer made the final decision.

A total of 1811 articles were retrieved, with all additional literature search system parameters set to their default states. The types of studies included in our selection are as follow:

(1) the year of publication is between 2009 and 2023; (2) only “article” and “review” type of documents are considered; (3) the selected language is English.

**Figure 1:**
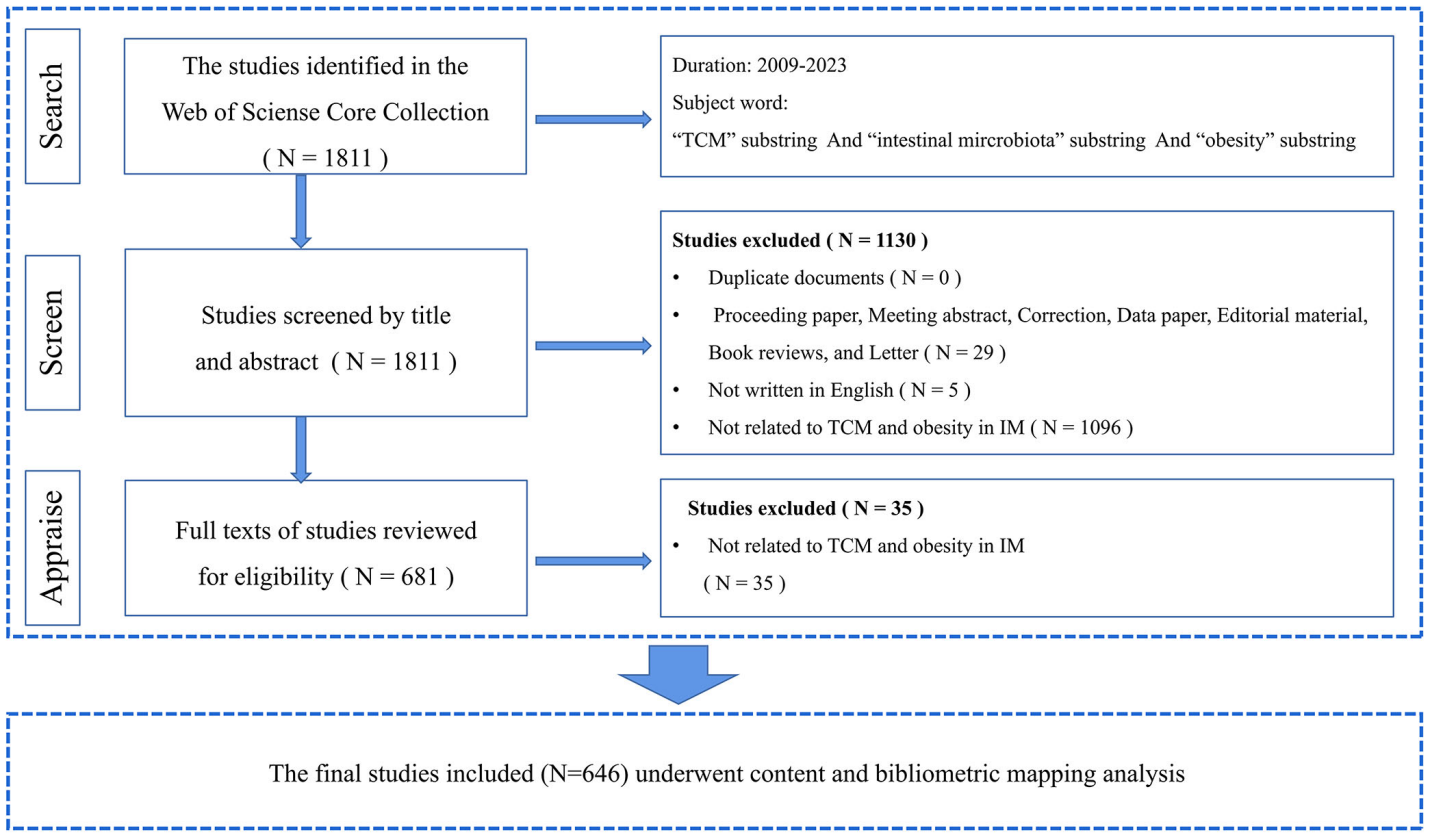
Screening flow chart.

The exclusion criteria are: (1): duplicated literature; (2) selection of literature that doesn’t align with the study content; (3) types of literature like proceedings papers, editorial material, letters, meeting abstracts, book reviews, corrections, data papers. Eventually, 646 pieces of literature were included. Detailed information on enrolment and selection is shown in **Figure 1**. All data collection and processing (including title, keywords, authors, country, institution, year of publication, h-index, and total citation count) was carried out by two authors (WH and JW). From January 1, 2009, to June 17, 2023, a total of 1811 documents related to the research subject were found via the search formula, finally leaving 646 pieces after manual filtering. Data presentation, analysis, and description were performed using Microsoft Excel 2016, VOSviewer version 1.6.19, CiteSpace version 6.2.R2, and the Online Analysis Platform (http://bibliometric.com/).

### 2.2 Bibliometric analysis

The knowledge graph is created using CiteSpace and VOSviewer. Both software have their own advantages, and they can complement each other. CiteSpace uses a data normalization method based on set theory to measure the similarity of knowledge units. The similarity algorithms generate timezone views and timeline views within time slices, thereby clearly delineating the evolution of knowledge and the historical span of literature in a certain cluster in time dimensions, allowing for the understanding of the progress and trends in this field (Chen, 2006). On the other hand, VOSviewer uses a probability-based data normalization method and offers various visualization views in terms of keywords, co-organizations, co-authors, etc., including network visualization, overlay visualization, and density visualization. It creates graphics that are easy to interpret and visually appealing (Eck and Waltman, 2009). We also used the H-index as an objective measure for evaluating scientific achievements. The H-index represents the fact that a maximum of h articles by a researcher have been cited at least h times. The higher an individual’s H-index, the greater the impact of their papers (Hirsch, 2005). The impact factor (IF) was provided by Journal Citation Reports™ published in 2022. These factors have made huge contributions to the assessment of article quality, making them key indicators for article evaluation (Eyre-Walker and Stoletzki, 2013).

## 3 Results

In terms of results, based on the search strategy depicted in **Figure 1**, we gathered a total of 646 papers composed between 2009 and 2023. These papers involved 44 countries, 849 organizations and 4255 authors. The publications were distributed across 184 journals and received a cumulative citation of 14,572 times, providing an average citation per paper of 22.56 instances. The H-index for the entirety of the publications stood at 60.

### 3.1 Annual trends in the number of publications

**Figure 2:**
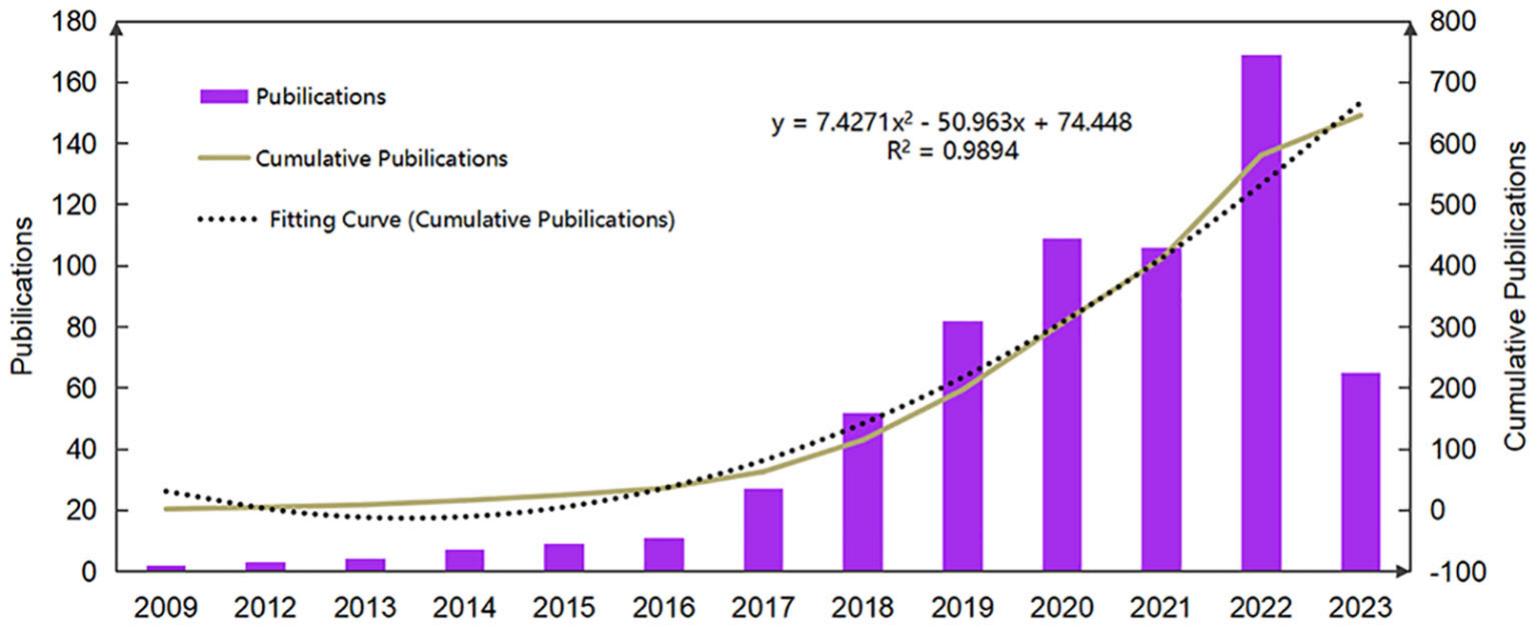
Publication growth trend of IM and obesity in TCM from 2009 to 2023.

The annual publication count serves as an indicator of the pace of research progression and the interest level in this research area. The domain of TCM for mitigating obesity and related metabolic disorders via IM modulation has demonstrated a consistent growth trend from 2009 to 2023. From 2009 to 2016, a moderate increase in the annual number of publications was noted. The growth rate accelerated from 2017 onwards, surpassing 100 publications in 2020. Although 2021 witnessed a decline in the volume of publications, research in this field has been steadily escalating year by year (**Figure 2**). The publication volume curve aligns with the quadratic function curve, with a fit R²of 0.9894. This suggests an escalating scholarly interest in this area, establishing it as a novel research focus in TCM, predicting a swift publication growth in the future.

### 3.2 Country collaborative network

**Figure 3:**
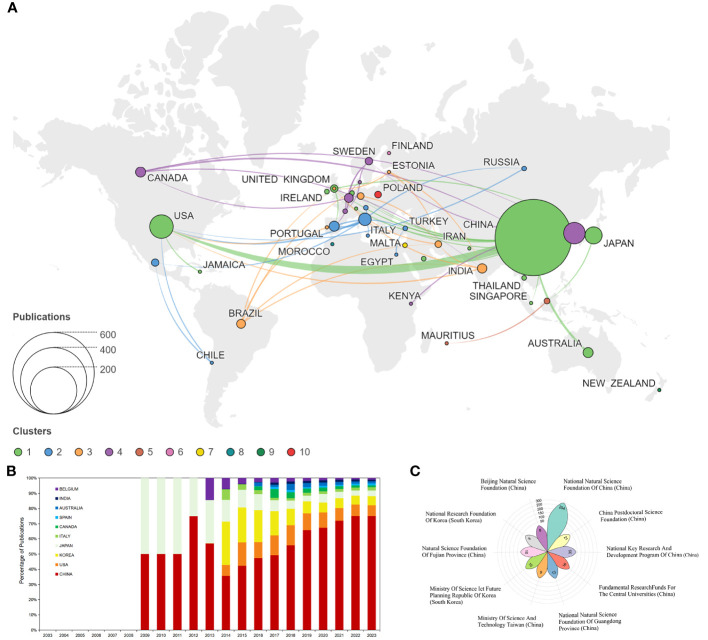
Analysis of countries publications. **(A)** Network diagram of collaborative relations between countries. Node sizes correspond to the number of publications, with distinct clusters indicated by different colors. The degree of cooperation is shown by the thickness of the connecting lines. **(B)** Percentage stacked area chart presents annual publications from the top 10 countries. **(C)** Publications originating from the top 10 foundations.

A publication analysis from various countries offers insights into a country’s significance and influence within a research field. Using VOS clustering, national collaboration networks were categorized into five clusters. Five of these are collaborative network clusters, while Finland, Israel, Morocco, New Zealand, and Poland do not collaborate with other countries (clusters 6 to 10, respectively). The distribution of national publications across different collaborative network clusters is notably uneven, with a pronounced top effect, and the majority of papers are contributed by scholars from a select few countries. Collaboration between China and the United States is more frequent, while collaborations amongst other countries are generally less frequent, suggesting a need for deeper international collaboration to advance the discipline (**Figure 3A**). The percentage stacking area plot indicates the relative trend of annual publications from the top 10 countries. Publications from China and Japan emerged relatively early, beginning in 2009. Publications from Belgium appeared in 2013, while those from the United States, Korea, and Italy emerged around 2014. Canada, Australia, Spain, and India joined later around 2016-2017 (**Figure 3B**). These findings indicate growing research interest in TCM for IM and obesity, heralding a rapidly expanding field (**Table 1**). lists the top 10 most productive countries. China leads with 518 papers, accounting for 80.19% of total publications, followed by the United States (49, 7.59%) and South Korea (41, 6.35%). China also dominates in total citations (11121, 76.32%), followed by the United States (2716, 18.64%) and South Korea (725, 4.98%). Belgium topped the average citation count (62.71), with the United States (55.43) and Canada (40.67) trailing behind. This suggests high-quality publications from these three countries, while the relatively low average citation rates in South Korea and India suggest room for quality improvement in these countries. The funding information in this field reveals that the Chinese Natural Science Foundation funded the most projects (294, 45.51%), followed by the National Key Research And Development Program Of China (35, 5.42%) (**Figure 3C**). The China Postdoctoral Science Foundation (21, 3.25%) ranked third. This reflects China’s substantial support in this research field.

**Table 1:** The top 10 most productive countries.

Rank	Country	Np	TC	H-index	Average citations
1	CHINA	518	11121	52	21.47
2	USA	49	2716	23	55.43
3	SOUTH KOREA	41	725	14	17.68
4	JAPAN	26	634	14	24.38
5	ITALY	14	317	7	22.64
6	CANADA	9	366	8	40.67
7	SPAIN	9	260	5	28.89
8	AUSTRALIA	9	357	9	39.67
9	INDIA	8	153	5	19.13
10	BELGIUM	7	439	5	62.71

### 3.3 Organizations and author analysis

**Figure 4:**
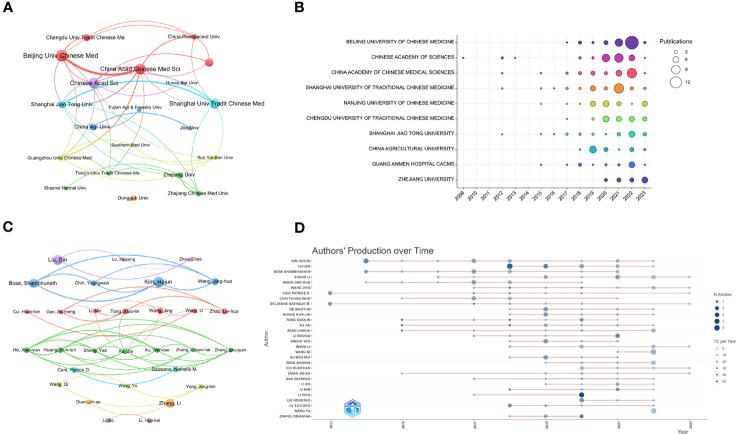
**(A)** The co-occurrence network of the top 20 corresponding organizations. The size of each node corresponds to the number of publications, while different colors denote distinct clusters. The line thickness demonstrates the extent of cooperation. **(B)** The top 10 most productive organizations over time, with the circle sizes proportional to the quantity of their publications. **(C)** The co-occurrence network of the top 30 authors of annual scientific publications. **(D)** The top 20 most productive authors over time, with the circle sizes reflecting the volume of their publications, and the color gradient signifying the annual number of citations.

Over 800 organizations contributed to this study, with the top 10 most productive ones listed in (**Table 2**). Beijing University of TCM leads with 34 publications, followed by the Chinese Academy of Sciences with 32, and the China Academy of Chinese Medical Sciences ranking third with 31. In terms of average citation rate, Guang An Men Hospital CACMS tops the list (*51.24*), followed by Shanghai Jiao Tong University (*41.91*) and China Academy of Chinese Medical Sciences (*35.55*). The organizational collaboration co-occurrence map depicts the collaborative relationships among the top 20 contributing organizations, categorized into seven clusters by VOS. Among these, Beijing University of TCM exhibits the strongest collaborative relationship with the Chinese Academy of Chinese Medical Sciences. However, collaborations outside these clusters are weaker, suggesting a need for more extensive collaboration (**Figure 4A**). Among the top 10 organizations in terms of annual scientific productivity, Beijing University of Chinese Medicine has the most publications in 2022 (**Figure 4B**).

**Table 2:** The top 10 most productive organizations.

Rank	Organizations	Country	Np	TC	H-index	Average citations
1	BEIJING UNIVERSITY OF CHINESE MEDICINE	China	34	692	13	20.35
2	CHINESE ACADEMY OF SCIENCES	China	32	890	14	27.81
3	CHINA ACADEMY OF CHINESE MEDICAL SCIENCES	China	31	1102	15	35.55
4	SHANGHAI UNIVERSITY OF TRADITIONAL CHINESE MEDICINE	China	30	722	15	24.07
5	NANJING UNIVERSITY OF CHINESE MEDICINE	China	24	462	14	19.25
6	CHENGDU UNIVERSITY OF TRADITIONAL CHINESE MEDICINE	China	23	294	9	12.78
7	SHANGHAI JIAO TONG UNIVERSITY	China	22	922	12	41.91
8	CHINA AGRICULTURAL UNIVERSITY	China	19	460	10	24.21
9	GUANG ANMEN HOSPITAL CACMS	China	17	871	9	51.24
10	ZHEJIANG UNIVERSITY	China	17	292	10	17.18

The co-occurrence network of the top 30 authors’ annual research contributions is displayed in (**Figure 4C**). The evolution of the top 30 authors’ annual scientific productivity over time is also summarized in (**Figure 4D**). Most authors published their papers since 2014, with the most influential papers appearing in 2015. The top 10 most productive authors are listed in **Table 3**. They produced 90 publications, garnering 2726 citations, which account for 13.76% of total citations. Among these prolific authors, Liu, Bin stands out with 14 publications and 495 citations from 2018 to 2022. His highly cited article supports the notion that Ganoderma triterpenoids inhibiting hepatic lipid accumulation and steatosis could be a potential novel functional food for treating or preventing hyperlipidemia (Guo et al., 2018). The second most prolific author is Kim, Hojun, with 12 publications and 470 citations from 2014 to 2022, followed by Bose, Shambhunath, who also has 12 publications, but with 351 citations. Both scholars, affiliated with Dongguk University, have collaborated frequently. An analysis of their published works indicates a keen interest in IM mechanisms, such as the intestinal mucosal barrier (Wang et al., 2014), bile acids metabolism (Han et al., 2017) and metabolic phenotype (Lee et al., 2014). Additionally, Tong, Xiao-lin with the highest average citation rate of 85.38, and his article demonstrated through a randomized, double-blind, placebo-controlled clinical trial for diabetes mellitus that the Gegen Qinlian Decoction could lower glucose by regulating IM changes (Xu et al., 2015).

**Table 3:** The top 10 most productive authors.

Rank	Authors	Np	TC	H-index	Average citations
1	Liu,Bin	14	495	11	35.36
2	Kim, Hojun	12	470	11	39.17
3	Bose, Shambhunath	12	351	9	29.25
4	Wang, Jing-hua	9	252	8	28
5	Tong,Xiao-lin	8	683	7	85.38
6	Cani, Patrice D.	7	471	6	67.29
7	He, Xiao-yun	7	171	6	24.43
8	Lv, Xu-cong	7	370	7	52.86
9	Zhao, Lin-hua	7	430	5	61.43
10	Delzenne, Nathalie M.	7	471	6	67.29

### 3.4 Main journals analysis

**Figure 5:**
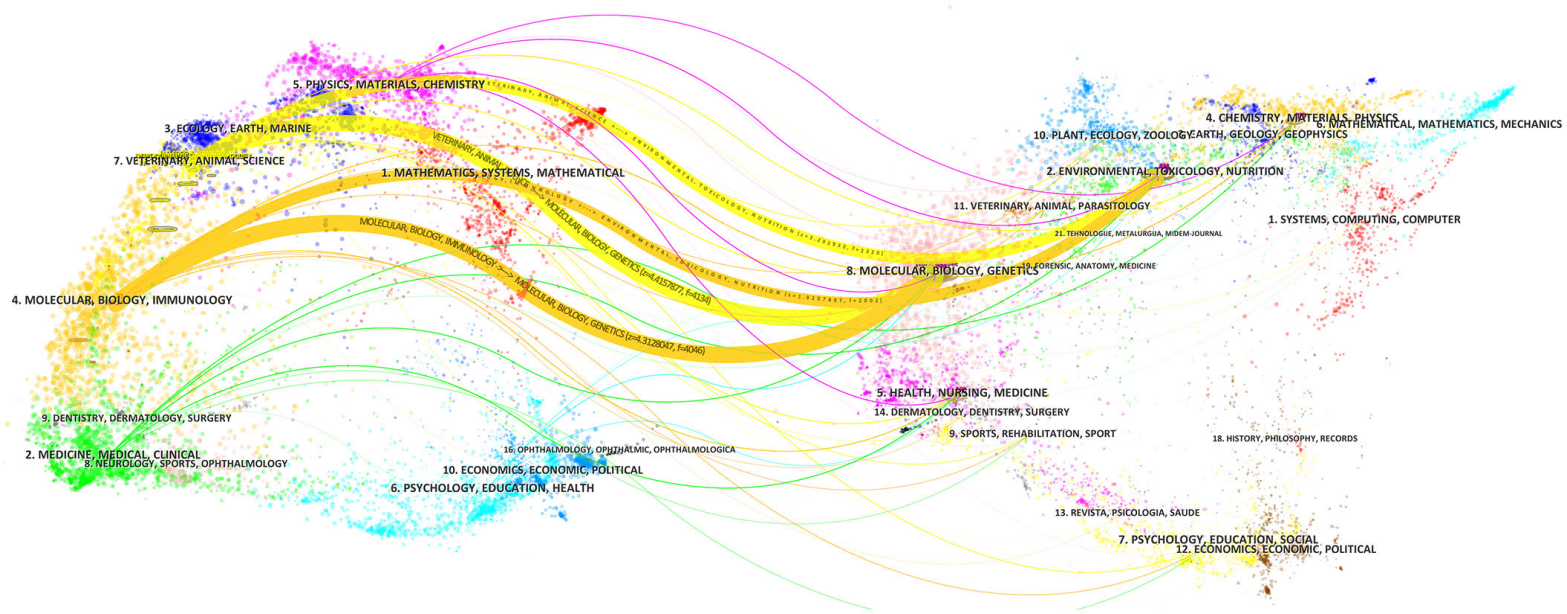
Dual map overlay of journals. The left side represents the fields of citing journals collection in the study, the right side represents the fields of cited journals collection, and the colored paths indicate the citation relationships.

The journals publishing the papers were tallied, with most journals in this field from 2009-2023 falling under the categories of Pharmacology, Nutrition and Microbiology, barring a few comprehensive journals. **Table 4** lists the top 10 journals based on the number of publications. Journals with over 20 articles include Food & Function, Frontiers in Pharmacology, Journal of Functional Foods, and Journal of Ethnopharmacology, with 41, 38, 28, and 28 articles respectively. Notably, Journal of Functional Foods, Frontiers in Pharmacology, and Journal of Ethnopharmacology are all open access journals, suggesting the robust growth of open access journals in recent years has significantly advanced research in this field. The analysis of journal citations revealed that Food & Function, with a total of 41 articles, an H-index of 18, and an average citation of 30.27, has the highest number of articles and average citations. This suggests that the journal has garnered significant attention in this paper’s research field. An examination of the published papers indicates that the journal primarily contains empirical research papers, focusing on food component chemistry, biochemical and physiological effects of food components, food nutrition aspects, toxicological responses to food components, and clinical and population studies using food or food components. A dual-map overlay analysis revealed the global science portfolio’s pattern on a global map of scientific journals. A dual map of journals on IM and obesity in TCM from 2009 to 2023 is depicted in (**Figure 5**). Each node on the map represents a journal, with the map divided into two parts: the left side represents citing journals, and the right side represents cited journals. All color curves on the map symbolize citation linkage lines, indicating the citation link path. The Z-scores function highlights a stronger, smoother trajectory, with a higher function value resulting in a thicker curve line and a stronger impact. As shown, veterinary, animal, science (yellow line, z=4.4157877, f=4134 and z=2.292933, f=2320) and molecular, biology, immunology (orange line, z=4.3128047, f=40463 and z= 4.4179473, f=2002) are influenced by environment, toxicology, nutrition, and molecular, biology, genetics.

**Table 4:** The top 10 most productive journals.

Rank	Journals	Np	TC	H-index	IF	Partition	Average Citation
1	FOOD & FUNCTION	41	1241	18	6.1	Q1	30.27
2	FRONTIERS IN PHARMACOLOGY	38	590	13	5.6	Q1	15.53
3	JOURNAL OF FUNCTIONAL FOODS	28	589	16	5.6	Q2	21.54
4	JOURNAL OF ETHNOPHARMACOLOGY	28	533	13	5.4	Q1	19.04
5	NUTRIENTS	25	438	11	5.9	Q1	17.52
6	BIOMEDICINE & PHARMACOTHERAPY	23	584	15	7.5	Q1	25.39
7	FRONTIERS IN NUTRITION	22	63	4	5.0	Q1	2.86
8	PHYTOMEDICINE	17	146	8	7.9	Q1	8.59
9	FRONTIERS IN MICROBIOLOGY	14	311	5	5.2	Q1	22.21
10	MOLECULAR NUTRITION FOOD RESEARCH	13	389	9	5.2	Q1	29.92

### 3.5 Reference analysis

**Figure 6:**
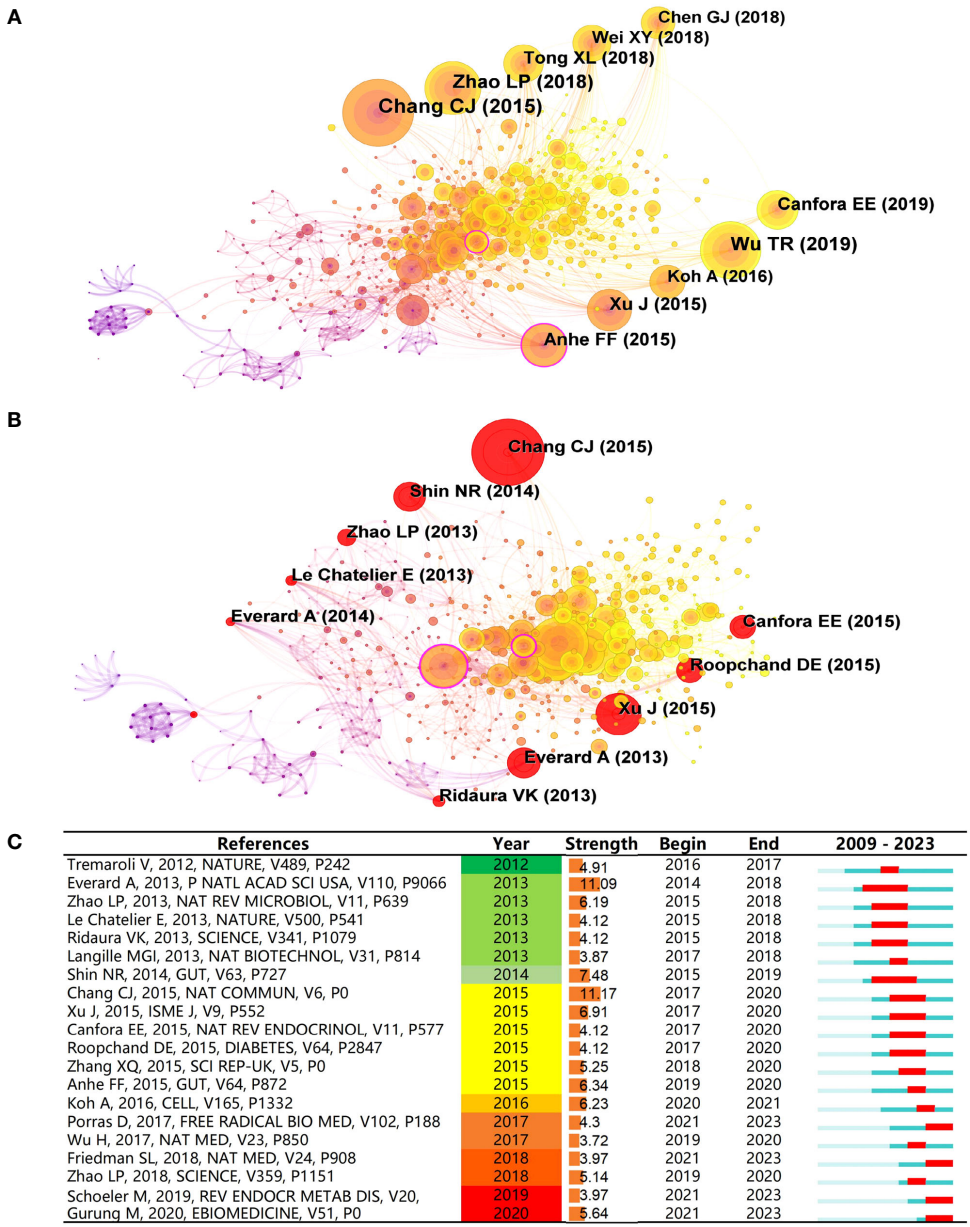
Reference co-citation network analyse. Node size corresponds to the number of citations, and nodes outlined in purple denote high centrality. Links represent co-citation relationships, while the position and color of nodes from left to right reflect the year of the cited reference. **(A)** Co-citation analysis of references. **(B)** Burst detection of co-citations, red nodes represent bursts. **(C)** Burst detection analysis for the top 20 references.

The core feature of CiteSpace is reference co-citation analysis (Synnestvedt et al., 2005). Reference co-citation refers to the occurrence of two references being jointly cited by one or more later papers, with the set of applied citations forming the knowledge structure and research frontier associated with the clustering (Chen et al., 2012). Consequently, a cluster analysis of references aids in understanding the field’s core information and the research’s evolution and development (Chen et al., 2010). Therefore, we employed CiteSpace version 6.2.R2 and set a g-index of 25, which is standard in our research field. This approach enabled the extraction of 576 influential citations from the literature. The results can be seen in **Figure 6A**, where the labels display the top 10 most cited publications, along with the first author and the year of publication. In order to measure the importance of key papers, we used two metrics: Local Citation Score (LCS) and Global Adoption Citation Score (GCS). LCS corresponds to a paper’s citations in the downloaded dataset, with a higher LCS indicating more attention from the field (**Table 5**). GCS indicates the number of times a paper is cited by all papers in the WoS database, suggesting attention from other fields and specialties to this literature (**Table 6**).

**Table 5:** The top 10 papers of high LCS.

Authors	Title	Journals	Type	LCS
Xu J, et al	Structural modulation of gut microbiota during alleviation of type 2 diabetes with a Chinese herbal formula	ISME J	Clinical Trial	49
Wu TR, et al	Gut commensal Parabacteroides goldsteinii plays a predominant role in the anti-obesity effects of polysaccharides isolated from Hirsutella sinensis	GUT	Animal experiment	41
Shi LL, et al	MDG-1, an Ophiopogon polysaccharide, regulate gut microbiota in high-fat diet-induced obese C57BL/6 mice	Int J Biol Macromol	Animal experiment	18
Martel J, et al	Anti-obesogenic and antidiabetic effects of plants and mushrooms	Nat Rev Endocrinol	Review	17
Zhang YP, et al	Effects of shenling baizhu powder herbal formula on intestinal microbiota in high-fat diet-induced NAFLD rats	Biomed Pharmacother	Animal experiment	16
Cao Y, et al	Modulation of Gut Microbiota by Berberine Improves Steatohepatitis in High-Fat Diet-Fed BALB/C Mice	Arch Iran Med	Animal experiment	15
Sun SS, et al	An insoluble polysaccharide from the sclerotium of Poria cocos improves hyperglycemia, hyperlipidemia and hepatic steatosis in ob/ob mice via modulation of gut microbiota	Chin J Nat Med	Animal experiment	15
Chen F, et al	Could the gut microbiota reconcile the oral bioavailability conundrum of traditional herbs?	J Ethnopharmacol	Review	14
Kim BS, et al	The anti-obesity effect of Ephedra sinica through modulation of gut microbiota in obese Korean women	J Ethnopharmacol	Clinical Trial	13
Song MY, et al	Influence of Panax ginseng on obesity and gut microbiota in obese middle-aged Korean women	J Ginseng Res	Clinical Trial	13

**Table 6:** The top 10 papers of high GCS.

Authors	Title	Journals	Type	GCS
Chang CJ, et al	Ganoderma lucidum reduces obesity in mice by modulating the composition of the gut microbiota	Nat Commun	Animal experiment	699
Wu TR, et al	Gut commensal Parabacteroides goldsteinii plays a predominant role in the anti-obesity effects of polysaccharides isolated from Hirsutella sinensis	GUT	Animal experiment	383
Xu J, et al	Structural modulation of gut microbiota during alleviation of type 2 diabetes with a Chinese herbal formula	ISME J	Clinical Trial	306
Tsuda T, et al	Curcumin as a functional food-derived factor: degradation products, metabolites, bioactivity, and future perspectives	Food Funct	Review	210
Tong XL, et al	Structural Alteration of Gut Microbiota during the Amelioration of Human Type 2 Diabetes with Hyperlipidemia by Metformin and a Traditional Chinese Herbal Formula: a Multicenter, Randomized, Open Label Clinical Trial	MBIO	Clinical Trial	199
Martel J, et al	Anti-obesogenic and antidiabetic effects of plants and mushrooms	Nat Rev Endocrinol	Review	170
Zeng SL, et al	Citrus polymethoxyflavones attenuate metabolic syndrome by regulating gut microbiome and amino acid metabolism	Sci Adv	Animal experiment	166
Neyrinck AM, et al	Polyphenol-rich extract of pomegranate peel alleviates tissue inflammation and hypercholesterolaemia in high-fat diet-induced obese mice: potential implication of the gut microbiota	Br J Nutr	Animal experiment	165
Axling U, et al	Green tea powder and Lactobacillus plantarum affect gut microbiota, lipid metabolism and inflammation in high-fat fed C57BL/6J mice	Nutr Metab	Animal experiment	160
Lopresti AL, et al	The Problem of Curcumin and Its Bioavailability: Could Its Gastrointestinal Influence Contribute to Its Overall Health-Enhancing Effects?	Advances In Nutrition	Review	151

#### 3.5.1 Local citation score and global adoption citation score

The LCS ranked first shows that the most cited reference is a clinical study published in “Randomized Controlled Trial” in 2015. This study demonstrated, through a double-blind clinical trial, that Gegen Qinlian Decoction (GQD) can regulate the composition of IM in treating T2D, with a significant increase in beneficial bacteria such as Faecalibacterium spp. These changes in the IM are related to the anti-diabetic effect of GQD (Xu et al., 2015). The second one is an animal experimental study published in Gut in 2019. This study discovered that high molecular weight polysaccharides derived from Trichoderma sinensis mycelium (HSM) stimulate the proliferation of Parabacteroides goldsteinii. Confirming this effect through fecal microbiota transplantation (FMT), the study revealed a significant reduction in weight gain and a marked improvement in metabolic disorders in mice consuming a high-fat diet. These findings suggest the potential of HSM polysaccharides and P. goldsteinii to serve as innovative prebiotics and probiotics, respectively, thus introducing new possibilities in obesity treatment (Wu et al., 2019). The third one is an animal experimental study published in the International Journal of Biological Macromolecules in 2015, which shows that MDG-1, a water-soluble β-d-fructan extracted from the roots of Ophiopogon japonicus, regulates IM such as decreasing the ratio of Firmicutes/Bacteroidetes, adjusting the abnormal IM to the normal state and altering their metabolic profiles (Shi et al., 2015). In addition, the most cited article in GCS was published in Nature Communications in 2015. The study proves that the water extract of Ganoderma lucidum mycelium (WEGL) can reverse not only HFD-induced gut dysbioses, such as reducing the ratio of Firmicutes to Bacteroidetes and levels of endotoxin-carrying Proteobacteria, but also maintain intestinal barrier integrity and reduce metabolic endotoxemia, and its anti-obesity and microbiota-regulating effects can be transplanted to HFD-fed mice through fecal microbiota transplantation (Chang et al., 2015). All four articles emphasize the significance of TCM regulation of IM in improving obesity. Overall, the most cited references listed in **Table 5** have made significant contributions to elucidating the connection between TCM, IM and obesity in the scientific community, earning their recognition as key references in this field.

#### 3.5.2 High centrality of references

The nodes with high centrality are within a cluster or between two clusters and are used to connect different articles or clusters within a cluster and facilitate the shift of research topics and research paradigms (Linton, 1978; Brandes, 2001). The node with the highest centrality is an animal experiment published in Gut in 2015, located in cluster #2 akkermansia muciniphila with a centrality of 0.14. The research unveils that polyphenols exert beneficial metabolic impacts on the characteristics of metabolic syndrome invoked by the high-fat/high-sucrose mice model, relating to the proportional increase of Akkermansia spp (Anhê et al., 2015a). The article was cited most in cluster #4 obese middle-aged Korean women, which found a correlation between herbal medicine and Akkermansia muciniphila, promoting the research development of IM in the obese populace through TCM. The second paper was published in 2016 in Nature Review Endocrinology, located in cluster #0 traditional Chinese medicine, holding a centrality of 0.11. The paper summarizes the anti-obesogenic and antidiabetic effects of plants and fungi in TCM. A high citation zone is primarily confined within its cluster (Martel et al., 2017). The third paper is an animal experiment published in the Archives of Iranian Medicine in 2016, similarly located in cluster #2, with a centrality of 0.1. The study showed that berberine administration restored the relative levels of bifidobacteria fed with HFD and the Firmicutes/Bacteroidetes ratio, consequently improving body weight, serum lipid levels, and glucose metabolism, while reducing the expression levels of CD14, IL-1, IL-6, and TNF-α (Cao et al., 2016). Thus, it’s visibly comprehended that the aforementioned three papers in cluster #0 and cluster #2 play a significant role in the paradigm shift of this research field.

#### 3.5.3 Bursts of references

We further examined the bursts of references (**Figure 6B**), which serve to spotlight research hotspots—references that maintain a high-frequency occurrence for a specific period (Cotterill and Eglen, 2019). **Figure 6C** illustrates the bursts of the top 20 references concerning IM and obesity in TCM from 2009 to 2023. The time span starting from 2009 to 2023 is depicted by the blue line, whereas, the red line signifies the duration consistent with bursts citations. In recent years, there has been increasing interest in understanding the intricate relationship between dietary factors and the IM. A comprehensive review published in Reviews in Endocrine & Metabolic Disorders in 2019 explored the interactions between dietary lipids and IM, shedding light on their impact on host physiology. The article also delved into the fascinating realm of membrane lipids synthesized by the IM and their potential roles in host signaling pathways (Schoeler and Caesar, 2019). In 2020, a valuable review published in EBIOMEDICINE shed light on the association between IM and T2D. This comprehensive review thoroughly examined 42 clinical studies, revealing specific gut microbial species, including Bifidobacterium, Bacteroides, Faecalibacterium, Akkermansia, and Roseburia were found to be negatively correlated with T2D, suggesting that a decrease in their abundance may be associated with an increased risk of T2D. These findings not only contribute to our understanding of the link between IM and T2D but also offer important clues for further exploring the relationship between IM and obesity. Moreover, these insights could potentially shape future research directions, inform clinical interventions, and guide the development of personalized strategies for managing T2D and obesity (Gurung et al., 2020). Among these contributions, an animal experimental study from Nature Communication in 2015 holds the highest intensity at 11.17. Its reference bursts commenced in 2017 and concluded in 2020 (Chang et al., 2015). The second-ranked burst intensity is 11.09, it burst in 2014 and culminated in 2018, proves that Akkermansia muciniphila can reverse the metabolic disorders induced by a high-fat diet, including increased fat mass, metabolic endotoxemia, adipose tissue inflammation, and insulin resistance (Everard et al., 2013).

#### 3.5.4 Reference co-citation clustering

**Figure 7:**
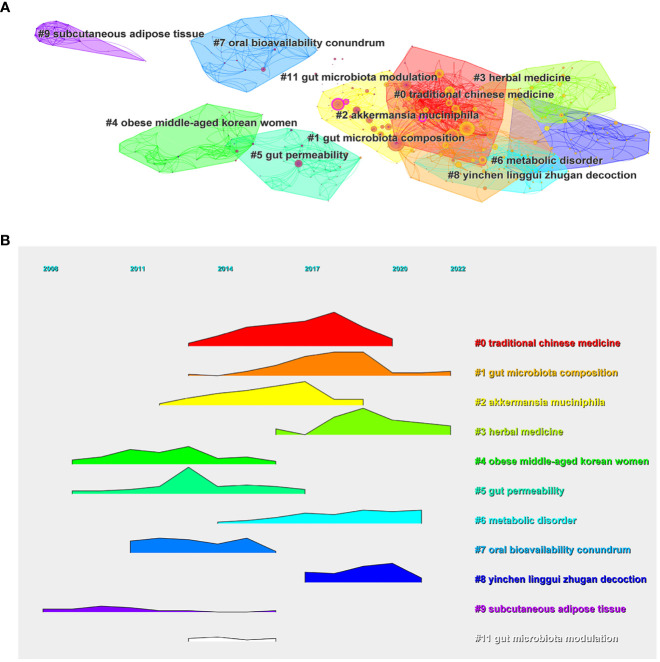
**(A)** The co-citation clustering network of references. **(B)** Ridgeline plot illustrating the co-citation clustering of references.

The scientific knowledge map of the reference co-citation clustering network was mapped from the title of cited references using the log-likelihood algorithm (LLR), and 12 clusters extracted from the references of 646 cited literature were thoroughly examined (**Figure 7A**). The 12 clusters include #0 traditional Chinese medicine, #1 gut microbiota composition, #2 akkermansia muciniphila, #3 herbal medicine, #4 obese middle-aged Korean women, #5 gut permeability, #6 metabolic disorder, #7 oral bioavailability conundrum, #8 yinchen linggui zhugan decoction, #9 subcutaneous adipose tissue, #11 gut microbiota modulation. Clusters where Q=0.6355 and S=0.8479 indicate reasonable clustering results, while cluster #10 did not form clustering links, suggesting this cluster is less relevant within the co-citation network and therefore is not discussed. The ridgeline plot illustrates the temporal dynamics of the various research themes (**Figure 7B**).

The clustering of subcutaneous adipose tissue and gut permeability studies, which started around 2008-2009, has gradually diminished, ceasing around 2015-2016. Conversely, the co-citation concerning IM composition and herbal medicine, initiated between 2013-2015, has remained steady, signifying their ongoing significance in the field. This thematic shift suggests the changing research trends in TCM-oriented IM and obesity studies. Initially focusing on pathophysiological aspects of obesity, such as subcutaneous adipose tissue and gut permeability, the field has progressively adopted a more holistic view, incorporating IM composition and herbal medicine interaction. This trend emphasizes the mounting acknowledgment of IM’s role in obesity and the prospective benefits of herbal medicine. The co-citation examination presents invaluable ideas about the gradual progress of IM and obesity research in TCM, shedding light on its principal themes and their timely progress. Consequently, it could steer future research paths and pinpoint unexplored areas in the existing corpus.

### 3.6 Keywords analysis

**Figure 8:**
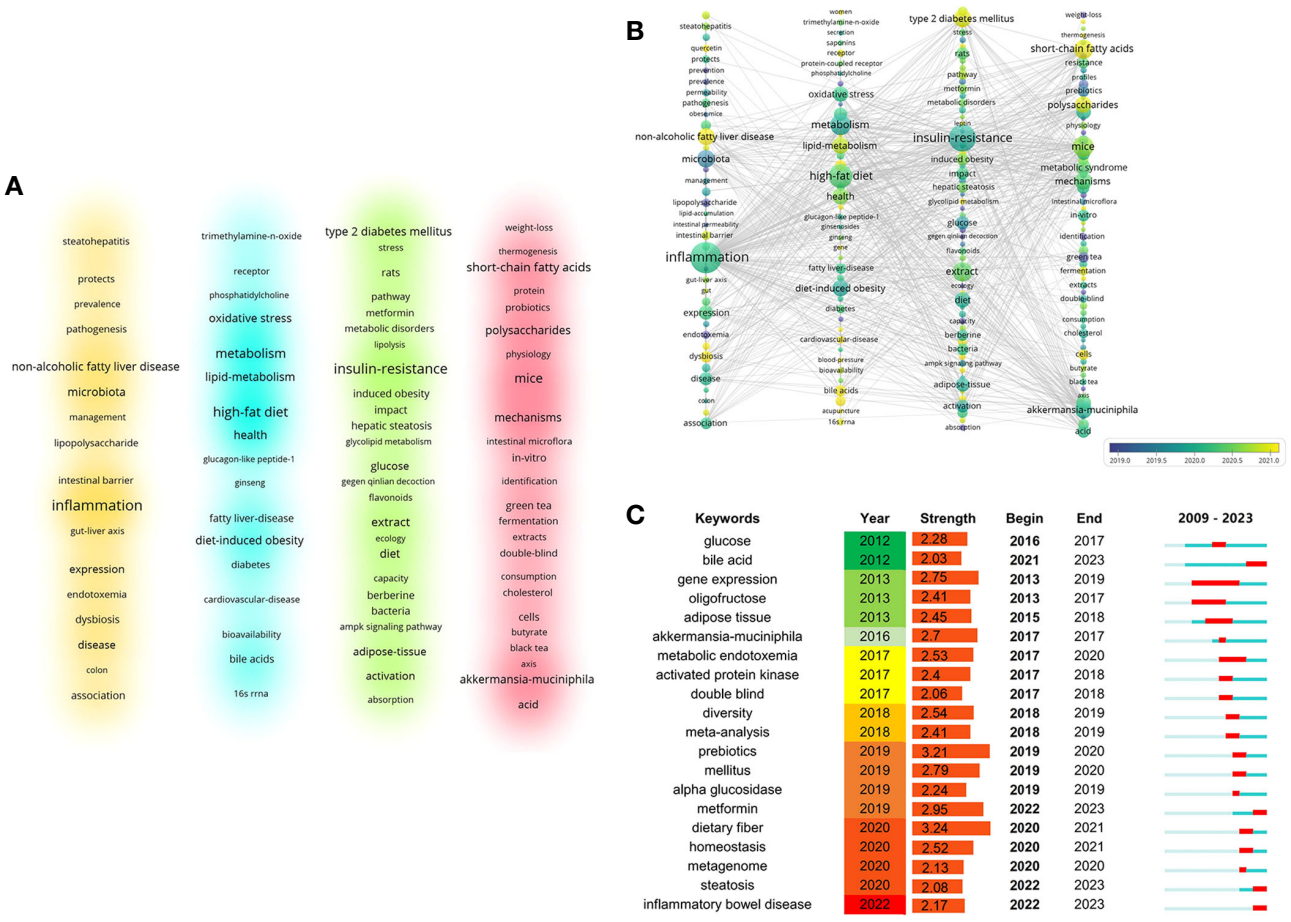
Keywords co-occurrence network analysis. **(A)** Keywords density visualization, with different colors representing distinct clusters. **(B)** Keywords co-occurrence and clustering overlay map,node sizes correspond to the frequency of the keywords, links symbolize co-occurrence relationships, and color signifies the average year of publications. **(C)** Burst detection analysis for the top 20 keywords.

A keyword co-occurrence and cluster analysis of 646 papers’ keywords (including author keywords and keyword plus) was conducted in VOSviewer (**Figure 8A**). High-frequency keywords (excluding search terms and little real significant keywords) include inflammation, insulin-resistance, high-fat diet, metabolism, short-chain fatty acids.The clustering is as follows:

1. The metabolic benefits mediated by endogenous functional metabolic molecules produced by TCM regulation of the IM. (Keywords: mice, mechanisms short-chain fatty acids, mechanisms, akkermansia-muciniphila, polysaccharides, polyphenols, antioxidant, metabolic syndrome, butyrate, fecal microbiota transplantation, bifidobacteria).
2. The metabolic benefits mediated by TCM restoration of the intestinal mucosal barrier. (Keywords: adipogenesis, barrier function, berberine, alkaloids, fatty liver disease, gegen qinlian decoction, glucose-metabolism, glycolipid metabolism, leptin, nf-kappa-b, ppar-gamma, tnf-alpha).
3. The metabolic benefits mediated by acupuncture regulation of the IM and the IM transformation mechanism of TCM herbals small molecules. (Keywords: 16s rrna, acupuncture, bile-acid metabolism, bioavailability, blood-pressure, cardiovascular disease, dyslipidemia, clinical trial, compound k, fxr, ginseng, ginsenosides, oxidative stress, glucagon-like peptide-1).
4. The mechanism of TCM regulation of the gut-organ axis. (Keywords: association, brain, colon, curcumin, gut-liver axis, intestinal barrier, endotoxemia, lipopolysaccharide, non-alcoholic fatty liver disease).

In VOSviewer, different research fields are visualized according to the color coding based on the average publication year (**Figure 8B**). Compared to earlier research keywords such as lipopolysaccharide (LPS) and endotoxemia, areas like bile acids (BAs), non-alcoholic fatty liver disease (NAFLD), acupuncture, short chain fatty acids (SCFAs), and cells have now become the main research fields in this domain. Meanwhile, inflammation, metabolism, oxidative stress, insulin-resistance, and akkermansia-muciniphila have emerged as research hotspots.20 keywords that appropriately represent this research field in terms of burst intensity, burst duration, and burst time (**Figure 8C**). From the analysis, it is evident that “dietary fiber” is the most frequently occurring keyword in this research field, with a burst intensity of 3.24. The burst for this keyword started in 2020 and lasted for one year. “Prebiotics” is the second most significant keyword with a burst intensity of 3.21, and its burst occurred between 2019 and 2020. Notably, dietary fiber, although a major component of herbal medicines, is usually not absorbed from the intestines into the bloodstream. Instead, it is often utilized as a prebiotic to regulate the intestinal microecology and improve host metabolism.In the early studies, the keyword “bile acid” appeared in 2012, but its burst continued from 2021 to the present. Bile acid is a vital metabolite in the gut-hepatic axis, known for its role in regulating energy metabolism and immune function through receptors like farnesol X receptor (FXR) and TGR5. Current research suggests that Chinese herbal medicine can modulate the metabolites produced by bile acids, such as cholic acid, goose deoxycholic acid, and porcine cholic acid, which, in turn, promote dietary fat solubilization and absorption (Lin et al., 2019; Deng et al., 2022).

## 4 Discussion

### 4.1 Research overview and characteristics of publications

In this paper, we used visualization and bibliometric analysis to identify the current status of research on the correlation between the regulation of IM by TCM and the prevention and treatment of obesity, intuitively displaying the annual number of publications, countries, organizations, journals, authors, references, keywords. This approach enables us to visually illustrate the system, research hotspots, and trajectory of TCM in regulating IM to prevent and treat obesity, thereby highlighting the research focus in this field. According to the SCI-E database of WoSCC, as of June 17, 2023, a total of 646 papers and reviews published from 2009 to 2023 were retrieved. Analyzing the cited references indicates a shift in the development trend of this field, moving from focusing on gut permeability and subcutaneous adipose tissue to studying microbiota composition and Chinese herbal medicinal ingredient. Dietary fiber and prebiotics are the keywords with the highest burst intensity in the past decade, reflecting the current research hotspots, with the keywords “bile acid, metformin, steatosis, and inflammatory bowel disease” further emphasizing this focus.

The changes in annual trends based on published papers demonstrate that research in this field has been continuously emphasized by researchers over the past ten years. From 2009 to 2016, the growth of publications was relatively slow, which was the initial stage of research. However, with the rapid development of high-throughput sequencing, 16S rRNA detection technology, and bioinformatics methods, the pace of publication in this field has accelerated since 2017 (Caporaso et al., 2010; Qin et al., 2012). Perhaps affected by the COVID-19 pandemic, the number of publications in 2021 has decreased (Gao et al., 2021), but the cumulative number of papers in this research field is still increasing year by year. Research in this field will continue to be a trend for a long time, and the number of papers will continue to surge.

Research indicates that papers related to IM and obesity in TCM primarily originate from China, followed by the United States, South Korea, and Japan. However, papers hailing from the United States and Belgium exhibit higher citation counts, suggesting the exceptional quality of these publications and their considerable value as references. The national collaboration network suggests more frequent collaboration between China and the United States, while past collaboration with other countries has been less. To foster progressive research in this field and address persistent issues, we strongly encourage scholars worldwide to transcend academic borders, engage in active discourse, and embrace collaboration.

The top ten institutions with the highest volume of publications are all in China, showcasing China’s excellent research capabilities in TCM. This is inseparable from the long history of TCM application in China and the high attention from the Chinese government, as well as the funding from projects like the National Natural Science Foundation. Among them, the Beijing University of Chinese Medicine has the highest volume of publications, the Chinese Academy of Sciences is ranked second, and the China Academy of Chinese Medical Sciences has the highest TC. The China Academy of Chinese Medical Sciences and Shanghai University of TCM have the highest H-index, with the Nanjing University of Chinese Medicine ranking second. These are top-notch research institutions in TCM in China, contributing significantly to IM and obesity in TCM.

The author with the highest number of publications is Liu Bin from Fujian Agriculture and Forestry University, followed by Kim Hojun and Bose Shambhunath from Dongguk University. The co-authorship network (**Figure 4C**) shows that there is less collaboration among authors, and the main researchers have not formed a collaboration network. It is suggested to strengthen academic exchanges among scholars, effectively enhance cooperation, and accelerate research progress in this field.

Using VOSviewer to analyze the papers published in this research field, 646 articles are distributed across 184 journals. FOOD & FUNCTION (IF: 6.1, JCR: Q1) is the most prolific in this research area. Among the top ten journals, three have impact factors exceeding 6, indicating that more in-depth or innovative research can be published in this field. The journal overlay dual-map shows the distribution of this study in different journal fields and the citation relationships between different journal fields. **Figure 5** shows that more research related to IM and obesity was published in the fields of Veterinary/Animal/Science and Molecular/Biology/Immunology. In addition, articles related to this study are also published in other fields, such as Physics/Materials/Chemistry, Ecology/Earth/Marine, Psychology/Education/Health. Articles related to this study in the fields of Veterinary/Animal/Science and Molecular/Biology/Immunology mainly focus on the specific mechanisms of TCM in regulating IM and preventing and treating Obesity through animal experiments.

### 4.2 Research current status and trends

The combination of reference co-citation analysis and high-frequency keyword analysis has shed light on the research hotspots within the field of IM and obesity in TCM. These hotspots primarily revolve around mechanisms of TCM in modulating IM and the metabolic benefits driven by endogenous functional metabolic molecules generated by TCM; Mucosal barrier restoration and gut-organ axis interaction; acupuncture’s impact on IM, and clinical research in TCM.

#### 4.2.1 Mechanisms of TCM in modulating IM and ameliorating obesity

##### 4.2.1.1 Modulation of IM composition and function

The interaction between the IM and TCM is a complex but very important research field. The IM can not only affect the absorption and metabolism of TCM, but may also affect the effects of TCM. Conversely, TCM can also influence the composition and function of the IM. TCM in regulating IM has shown promising effects in improving obesity and metabolic disorders by regulating the composition and function of the IM. Studies have demonstrated that TCM interventions can effectively modify the structure of the IM by increasing the abundance of beneficial bacteria and decreasing harmful bacteria (Chen et al., 2018; Shu et al., 2023). For instance, The ethanol extract of propolis, for instance, has been found to increase anti-obesity and anti-inflammatory bacteria such as genera Roseburia, Intestinimonas, species Parabacteroides goldsteinii, and Parabacteroides distasonis, while reducing pro-inflammatory bacteria like genera Faecalibaculum, Prevotella, and species Bacteroides vulgatus in high-fat diet (HFD) mice (Cai et al., 2020).

On the other hand, the IM can interact with the ingredients of TCM herbals, thereby affecting the therapeutic effect. For instance, the anti-obesity efficacy of Ophiocordyceps sinensis and its anamorph Hirsutella sinensis contingent upon their interaction with IM, as validated via antibiotic treatment and fecal microbiota transplantation experiments. Further, neomycin-sensitive bacteria have been discerned as pivotal to the anti-obesity action (Wu et al., 2019).

In addition, the ingredients in TCM may indirectly regulate the IM by affecting the host’s physiological state. Some TCM herbals can inhibit inflammatory responses, thereby changing the composition of the IM and the function of the intestinal barrier; some TCM may affect the host’s energy metabolism, thereby affecting the composition of the IM.

##### 4.2.1.2 Anti-inflammatory responses

The IM plays a crucial role in inflammation through its association with lipopolysaccharides (LPS), which can activate immune cells and induce cytokine production. Besides, IM can influence metabolic processes by regulating inflammatory responses and active molecular signaling, which are considered fundamental elements in the pathogenesis of obesity (Rastelli et al., 2019). Polygonatum kingianum polysaccharides have been observed to activate PPARγ, inhibit LPS-TLR4/NFκB immune responses, reduce the Firmicutes to Bacteroides ratio (F/B ratio), promote adipocyte differentiation, attenuate adipocyte volume, alleviate hepatocyte steatosis, elevate adipokine expression, enhance insulin sensitivity, and mitigate insulin resistance (Gu et al., 2020). Moreover, research has illustrated the anti-inflammatory efficacy of rhubarb extract. It can increase the abundance of Akkermansia muciniphila, a beneficial gut bacterium, to modulate the IM, reduce the expression of inflammation-related genes, and decrease the number of macrophages in adipose tissue (Régnier et al., 2020).

##### 4.2.1.3 Mucosal barrier restoration and gut-organ axis interaction

The normal intestinal mucosal barrier is composed of mechanical, chemical, immune, and biological barriers, which can prevent harmful substances such as bacteria and endotoxins in the intestine from passing through the intestinal mucosa into other tissues, organs, and blood circulation. Research has found that a high-fat diet can alter the structure of the IM, increase the proportion of Gram-negative bacteria, lead to an increase in lipopolysaccharide production, and the imbalance of the microbiota proportion can also increase the permeability of the intestinal epithelium by inhibiting the expression of tight junction proteins between intestinal epithelial cells, promoting lipopolysaccharides to enter the blood, causing metabolic endotoxemia and inducing obesity (Cani et al., 2007a; Cani et al., 2007b). TCM can improve the intestinal environment and increase the stability of the IM, thereby improving obesity and metabolic diseases. Research shows that Ganoderma lucidum extract (WEGL) can reduce the weight, inflammation, and insulin resistance of mice on a high-fat diet. At the same time, WEGL can not only reverse the intestinal microbial imbalance induced by HFD, such as the decrease F/B ratio and the level of endotoxin-containing Proteobacteria but also maintain the integrity of the intestinal barrier and alleviate metabolic endotoxemia (Chang et al., 2015). Crocin-I alleviated intestinal microbial diseases, decreased the F/B ratio and the abundance of Proteobacteria, decreased the expression levels of inflammatory cytokines, up-regulated the expression of occludin, Muc1, and Muc2 in colon tissue, increased the levels of SCFAs in the colon, and improved intestinal barrier function and colon tissue inflammation (Xie et al., 2022). In addition, the IM can interact with other organs, such as the liver, brain, and immune system, through various signaling pathways (Anlu et al., 2019). TCM can modulate these gut-organ axes to improve overall health. For example, TCM can regulate the gut-liver axis by modulating bile acid metabolism and reducing liver inflammation, and it can influence the gut-brain axis by altering the production of neurotransmitters and modulating neuroinflammation (Agirman et al., 2021; Yoon et al., 2021).

##### 4.2.1.4 Improving oxidative stress

Oxidative stress occurs when an imbalance between oxidative and antioxidant processes in the body leads to neutrophil infiltration and increased secretion of proteolytic enzymes, resulting in intestinal microecological disorders. Superoxide dismutases (SOD) are crucial in protecting intestinal mucosal cells from oxygen radical damage and are among the most important antioxidants in the body, along with catalase (CAT), glutathione peroxidase (GSH-Px), and others. Intestinal bacteria can stimulate reactive oxygen species (ROS) production via intestinal epithelial cells, and notably, probiotics can induce antioxidant activity in these cells (Aviello and Knaus, 2018; Vona et al., 2021; Kunst et al., 2023). Extracts from Dendrobium nobile possess antioxidant properties that increase the activities of SOD, CAT, and GSH-Px in the skin, blood, liver, and brain, increase the F/B ratio in aging mice, enhance the abundance of probiotic bacteria Lactobacillus and Muribaculum, and reduce the presence of pathogenic bacteria like Staphylococcus, Corynebacterium, and Brevibacterium (Gao et al., 2022). Dihydrocurcumin regulates glucose intake, moderates SREBP-1C/PPAR-α/pAKT expression, and decreases levels of triglycerides, oxidative stress, and insulin resistance (Yu et al., 2018). Leaves of Tetrastigma hemsleyanum mitigate hepatic lipid accumulation and oxidative stress by modulating the expression of lipid synthesis/catabolism and oxidative stress genes (SREBP-1c/ACC-1/PPAR-α/PPAR-γ/Keap1/Nrf2), attenuating oxidative stress related to NAFLD in mice and reducing lipid accumulation and inflammation (Xiao et al., 2023). Poria cocos polysaccharides ameliorate hepatic damage, inflammation, and oxidative stress in mice with NASH through regulation of IM and the NF-κB/CCL3/CCR1 axis (Tan et al., 2022).

Furthermore, certain herbs, notably Poria, have demonstrated potential as prebiotics, aiding in the propagation of beneficial microbiota and mitigating oxidative stress. Empirical evidence suggests that the consumption of Poria polysaccharides leads to an augmented presence of butyric acid-producing Trichoderma spp. and Clostridium spp. in the cecum of obese mice.This, in turn, results in increased intestinal butyrate content, improved intestinal mucosal integrity, and enhanced β-oxidation of fatty acids, while also reducing nitrate production through the activation of the PPAR-γ pathway. Additionally, FMT experiments have demonstrated that the GM mediates the pro-health effects of Poria cocos. These findings hold significance in exploring the role of traditional Chinese medicine ingredients in microbiota regulation and health promotion (Sun et al., 2019). Besides, the Tianhuang formula can increase the abundance of Lactobacillus in the gut and liver and 5-Methoxyindoleacetate (5-MIAA), activate NF2, and reduce the expression of oxidative markers GCLC, NQO1, and TXR1 (Luo et al., 2022). Interestingly, TCM can produce effects similar to prebiotics even at doses much lower than those typically used for conventional prebiotics (Anhê et al., 2015b). As research progresses, it is no longer unusual for extracts of traditional Chinese medicine to exhibit “prebiotic” properties. Polysaccharides from foods and medicine homologous plants that contain β-glycosidic bonds, such as Ganoderma polysaccharides, Lycium barbarum polysaccharide, Codonopsis pilosula Nannf can be developed into characteristic prebiotics of these homologous plants of food and medicine (Lyu et al., 2017; Zhu et al., 2020); Polyphenolic substances such as cinnamon can promote the growth of beneficial bacteria such as bifidobacteria and lactobacilli, selectively inhibit the growth of pathogenic bacteria, optimize the structure of the gut microbiota, and regulate the balance of the gut microecology (Van Hul et al., 2018).

##### 4.2.1.5 Regulation of glucose and lipid metabolism

For instance, a study using streptozotocin (STZ) and high-fat diet (HFD)-induced T2D rat models demonstrated how the EtOAc extract from Sophora flavescens can counter lipid, amino acid, and carbohydrate metabolic abnormalities. These benefits appear to be closely related to modifications in the interplay between the gut microbiome and metabolites (Shao et al., 2020). Similarly, research has shown that Fubrick tea significantly modulates the serum metabolome by IM, particularly influencing the ‘caffeine metabolism’ pathway. Furthermore, it can rectify both IM dysbiosis induced by a high-fat diet and impairments in lipid metabolism and glucose tolerance (Jing et al., 2020). Miao sour soup lowers body weight and lipid levels, as well as levels of TNF-α and IL-6 in the intestinal mucosa while it elevates the levels of PYY and GLP-1 in the colonic tissues, and increases the expression of PYY and ZO-1 in the intestinal epithelial cells in obese rats (Zhou et al., 2023). Polygonatum kingianum regulates the abundance and diversity of the IM community by increasing the relative abundance of SCFA-producing bacteria. It furthers the recovery of the intestinal permeability barrier, prevents LPS from entering the circulation, reduces inflammation, and averts glucose and lipid metabolic disorders (Gu et al., 2020). Moreover, a study discovered that Dendrobium officinale regulated TG, TC, LDL-C, and HDL-C to varying degrees by controlling Ruminococcus and Oscillospira, in addition to influencing carbohydrate, amino acid, and energy metabolism functions through the correlation analysis of lipids and IM (Li X. et al., 2022).

#### 4.2.2 TCM improves obesity through the metabolites of IM

TCM can regulate IM and its metabolites, such as short-chain fatty acids (SCFAs) and bile acids (BAs), which are important factors influencing the therapeutic effects of TCM (Lyu et al., 2017; Wang et al., 2019). Lv Xucong’s team found that Ganoderic acid A intervention significantly regulates bile acid biosynthesis, fatty acid biosynthesis, amino sugar and nucleotide sugar metabolism, and inositol phosphate metabolism in a high-fat diet. It also modulates the mRNA levels of liver genes involved in fatty acid metabolism and bile acid homeostasis, increases levels of short-chain fatty acids in the gut, promotes bile acid excretion through feces, inhibits abnormal weight gain, reduces adipocytes, and alters hepatic lipid metabolism to reduce lipid metabolic disorders and improve IM imbalance (Guo et al., 2020).

##### 4.2.2.1 Bile acids metabolisms

Bile acids (BAs) are the main products of cholesterol metabolism, and the IM may influence metabolism by affecting BA composition. The interaction between the IM and BAs plays multiple roles in controlling glucose and lipid metabolism, energy balance, and obesity related pathways through the FXR-mediated regulation of BA signaling (Thomas et al., 2008; Wang et al., 2008; Giannini et al., 2022). Treatment with Astragaloside IV(AS-IV) has been shown to alleviates high-fat diet-induced metabolic disorders, especially hepatic steatosis. After AS-IV administration, the abundance of BSH-carrying bacteria significantly decreases, inhibiting the hydrolysis of taurine-β-muricholic acid (T-β-MCA) and increasing the level of intestinal T-β-MCA. T-β-MCA can inhibit the transcriptional activity of the intestinal Fxr gene, reduce the secretion of fiberblast growth factor 15 (FGF15), promote the release of intestinal GLP-1, and lower serum levels of neurotensin, thereby inhibiting hepatic lipogenesis and improving fatty liver (Zhai et al., 2022). Another study reported that psyllium husk regulates the expression levels of FXR and CYP7B1, as well as the levels of cholic acid, deoxycholic acid, lithocholic acid, and ursodeoxycholic acid, thereby controlling hepatic fat content in high-fat diet (HFD) mice and ameliorating hypercholesterolemia and non-alcoholic fatty liver disease (Deng et al., 2022).

##### 4.2.2.2 Short-chain fatty acids

Short-chain fatty acids (SCFAs), including acetates, propionates, and butyrates, are produced by the IM through the fermentation and degradation of non-digestible fibers and polysaccharides in the diet. They are considered potential metabolic targets for glucose metabolism and insulin resistance in preventing obesity/T2D. SCFAs can activate the free fatty acid receptor 3 (FFAR3) to stimulate the secretion of intestinal hormone PYY, enhance glucose in muscle and fat tissues, and create a feeling of satiety, thereby reducing food intake. They also activate the FFAR2 to promote the secretion of GLP-1, increase the secretion of insulin, and reduce the secretion of pancreatic glucagon to regulate blood glucose levels indirectly (Tolhurst et al., 2012). A study used an HFD-induced rat model to compare the effects of berberine and metformin on the IM, finding that both interventions significantly enriched the abundance of SCFA-producing bacteria (Zhang et al., 2015). In subsequent research, it was proven that berberine, as an active ingredient of Gegen Qinlian Decoction (GQD), can significantly alter the overall structure of the IM and enrich many butyrate-producing bacteria, including Faecalibacterium and Roseburia, thereby significantly increasing the levels of SCFAs in feces, relieving intestinal inflammation, and reducing blood sugar levels. In addition, the concentrations of serum pro-inflammatory cytokines in the islets and the expression of immune-related genes (including Nfkb1, Stat1, and Ifnrg1) significantly decreased after treatment (Xu et al., 2020). Other studies have reported similar benefits from the consumption of ginger, which increased the abundance of Bifidobacterium and SCFA-producing bacteria like Alloprevotella and Allobaculum (Wang et al., 2020). In addition, the Cordyceps guangdongensis lipid-lowering formula regulated the IM and SCFAs, affecting the expression of lipid metabolism-related genes mRNA, such as up-regulating Pnliprp1, Pnliprp2, and Ppard, and down-regulating Cidea and Cidec (Wang et al., 2022). Other studies have shown that Huang-Lian-Jie-Du-Decoction improves high blood sugar levels and insulin resistance in rats by increasing SCFAs and anti-inflammatory bacteria such as Parabacteroides, Blautia, Akkermansia, and reducing conditional pathogens such as Aerococcus, Staphylococcus, and Corynebacterium (Chen et al., 2018).

#### 4.2.3 Herbal medicine compounds transformed by IM in obesity

The complexity of the components in TCM often results in low oral bioavailability of most active ingredients. Consequently, interactions with the IM inevitably occur, becoming important targets for the action of TCM. A review article by Professor Lai Xinzhi’s team from Chang Gung University in Taiwan pointed out the close relationship between IM composition and the conversion of active metabolites from herbal components, which could significantly affect the therapeutic activity of TCM (Lin et al., 2021). For instance, the bioavailability of ginsenosides is low, but the metabolites transformed by IM may possess biological activity. Studies have shown that prebiotics can promote the proliferation of certain bacterial strains with glycoside hydrolysis capacity, thereby improving the biotransformation and bioavailability of primary ginsenosides in the body (Zhang X. et al., 2021). Compound K, which has higher potency and activity than ginsenoside Rb1, is formed by the IM from ginsenosides Rb1, Rb2, and Rc. Compound K is absorbed into the bloodstream, metabolized again in the liver to form a hydroxylated compound, and exerts anticancer, antidiabetic, and anti-inflammatory effects (Wang et al., 2011; Kim, 2018).

#### 4.2.4 Acupuncture regulating IM in obesity

Acupuncture, as a special method of TCM treatment, includes acupuncture, electroacupuncture, moxibustion, and drug fumigation, which have extensive clinical applications. It has a significant regulatory effect on the IM, and can benignly regulate the content of physiological flora and the ratio of imbalanced microbiota, thereby promoting the richness and diversity of the IM, correcting its abnormal metabolic function to accelerate energy consumption, achieving the effect of weight loss. Recent research has shown that long-term local mild temperature heat therapy can resist and treat obesity in an HSF1-dependent manner without affecting the central sympathetic nervous system and the immune system, improve insulin sensitivity and liver lipid deposition, and does not produce obvious side effects (Li Y. et al., 2022). However, current research on the interaction between moxibustion and IM in this field is relatively scarce, mostly focusing on electroacupuncture treatment. Research has proven that electroacupuncture promotes the expression of multiple mucosal innate immunity-related alpha-defensin genes such as Defa20, Defa41, and Defa5, rescuing dysbiotic cecal microbiota, reducing peripheral inflammation factors and alleviating glucose tolerance, playing a role in resisting obesity (Xia et al., 2022). Another study showed that electroacupuncture might change the proportions of Bilophila and Bifidobacterium by regulating the constituents of the functional metabolite of 3a,6a,7b-Trihydroxy-5b-cholanoic acid, Cholanoic acid, and L-Arginine (Si et al., 2022).

#### 4.2.5 Clinical research on the correlation between obesity and IM by TCM intervention

Currently, the treatment of symptom clusters under the guidance of TCM theory and Chinese herbal medicine often needs more specificity, and its biological basis needs to be clarified. However, in the function of regulating IM, it involves changes in a large number of endogenous substances, which can fully reflect the overall state of the patient, and the development of IM research provides very appropriate biomarkers for TCM syndromes. In the research of IM, biomarkers may be specific types or groups of microorganisms, or some metabolites produced by these microorganisms. For example, TCM constitution and symptoms are related to the specific composition of the IM. The discipline of TCM constitution (TCMC) inherits the idea of “varying from person to person” in Chinese medicine and divides people into nine categories (Qi, 2019). Phlegm-dampness constitution (PDC), one of the nine TCM constitutions, is considered a high-risk factor for obesity and its complications (Dong-Po et al., 2011). A clinical study showed that obese individuals with PDC had higher levels of Bacteroidetes than non-PDC subjects, and the Firmicutes/Bacteroidetes ratio was significantly reduced. This suggests that the characteristics of the IM can be used as biomarkers to differentiate obese individuals with PDC or non-PDC under the microscope (Shin et al., 2022). Also, some scholars have researched Chinese medicine symptoms and proved that Chinese medicine symptoms are related to the intestinal mucosal microbiota (Zhu et al., 2022). A clinical study showed that the differences in amino acid metabolism of the IM are related to different TCM syndromes of metabolic syndrome (MS) and their clinical manifestations. The difference between the qi-yin deficiency syndrome (QYLX) group and the phlegm-dampness syndrome (TSZE) group in terms of IM amino acid metabolism is significant: the metabolism of alanine, aspartate, and glutamate, as well as the biosynthesis pathways for valine, leucine, and isoleucine, are more abundant in the QYLX group. In contrast, in TSZE patients, the metabolism of tryptophan, the degradation of lysine, the degradation of valine, leucine, and isoleucine, and the metabolism of phenylalanine are enriched (Shang et al., 2022). In addition, a multicenter double-blind randomized controlled clinical trial confirmed the blood sugar-reducing effect of berberine on newly diagnosed patients with abnormal blood sugar, and the combined use of Bifidobacteria enhanced the potential of berberine to reduce blood sugar. This is the first clinical research report applying this treatment plan to patients with abnormal blood sugar. The research further revealed the changes in the composition and function of the IM after berberine treatment, providing a basis for the research on the mechanism of berberine’s blood sugar-lowering and the application of berberine as a safe and effective hypoglycemic drug in clinical practice (Ming et al., 2021).These clinical studies show that TCMC and TCM symptoms are related to changes in the IM. Making IM a biomarker will be helpful and even provide a basis for early diagnosis. Future research may explore these biomarkers more deeply, for example, verifying the predictive power of these markers through large-scale population studies, or gaining a deeper understanding of the role of these markers in disease onset and development through experimental research. This research may help us better understand the pathogenesis of obesity and related diseases, and may also help develop new prevention and treatment strategies (**Table 7**).

**Table 7:** Comparative evaluation of TCM and positive control with regard to obesity-related metabolic diseases.

Intervention	Research type	Subjects	Outcome	Changes in IM	Disease	Ref
a specifically designed herbal formula (AMC)	Clinical trial	Human	HOMA-β↑; FBG, PBG, HbA1c, HOMA-IR, TC, TG, LDL-C↓	Blautia, Faecalibacterium, Roseburia, Gemmiger, Coprococcus, Megamonas↑	T2DM with hyperlipidemia	(Tong et al., 2018)
Metformin			HOMA-β↑; FBG, PBG, HbA1c, TC, LDL-C↓	Blautia↑; Alistipes, Oscillibacter, Bacteroides↓		
Berberine	Animal experiments	Wistar rats	BW, Adiposity index↓	Blautia, Allobaculum, Bacteriodes, Butyricoccus, Lactobacillus↑; Clostridium XlVa, Flavonifractor, Lachnospiracea_inc, Roseburia, Clostridium XI↓	Obesity	(Zhang et al., 2015)
Metformin			BW, Adiposity index↓	Akkermensia, Prevotella, Lactobacillus, Blautia, Allobaculum, Bacteriodes, Butyricoccus↑; Clostridium XlVa, Flavonifractor, Lachnospiracea_inc, Roseburia, Clostridium XI↓		
Scutellaria baicalensis+Metformin	Animal experiments	LETO rats	BW, FBG, TC, HOMA-ISI↓	Lactobacillus, Bacteroides↑; Clostridium, Enterobacter↓	T2DM	(Han et al., 2017)
Metformin			BW, FBG, HOMA-ISI↓	/		
Psyllium husk	Animal experiments	C57BL/6J mice	BW, TC, TG, LPS, LDL/HDL ratio↓	Firmicutes, Bacteroidetes, Bilophila, Sutterella, Roseburia, Bacteroides, Faecalibacterium, Coprobacillus, Parabacteroides distasonis↑; F/B ratio↓	Obesity with hypercholesterolemia and NAFLD	(Deng et al., 2022)
Orlistat			BW, TC, TG, LPS, LDL/HDL ratio↓	Firmicutes, Bacteroidetes, Sutterella, Akkermansia↑; F/B ratio↓		
Polygonatum kingianum	Animal experiments	SD rats	HDL-C↑; BW, FBG, TG, LDL-C, FINS, HOMA-IR↓	Bacteroides, Oscillibacter, Allobaculum, Blautia, Phascolarctobacterium↑; F/B ratio, Proteobacteria, Lactobacillus, Psychrobacter↓	Metabolic syndrome	(Gu et al., 2020)
Simvastatin			HDL-C↑; BW, FBG, TG, LDL-C, FINS, HOMA-IR↓	Proteobacteria↑		
Jiang-Tang-San-Huang	Animal experiments	SD rats	HDL-C↑; FBG, TBA, TNF-α, IL-6, IL-1β, TC, TG, LDL, FFA, FINS, AST, ALT, Cr, BUN↓	Romboutsia, Lactobacillus, Turicibacter, Bacteroides, Bifidobacterium, Clostridium-sensu stricto 1↑; F/B ratio↓	T2DM	(Tawulie et al., 2023)
metformin			FBG, TBA, TNF-α, IL-6, IL-1β, FINS↓	/		
Fufang-zhenzhu-tiaozhi formula	Animal experiments	C57BL/6 mice	TC, TG, Scr, ACR, Urinary albumin/creatinine ratio, serum creatinine, 24h albuminuria, kidney/body weight ratio, IL-6↓	Bacteroidetes↑; F/B ratio, Weissella, Enterococcus, Akkermansia↓	Diabetic kidney disease	(Lan et al., 2023)
Losartan			IL-6, serum creatinine, urinary albumin/creatinine ratio, 24h albuminuria, kidney/body weight ratio↓	/		
Qijian mixture	Animal experiments	KKay mice	BW, FBG↓	Bacteroidetes↑; Firmicutes, Actinobacteria↓	T2DM	(Gao et al., 2018)
Metformin			BW, FBG, INS↓	Bacteroidetes, Proteobacteria↑; Firmicutes, Actinobacteria↓		
Scrophulariae Radix and Atractylodes sinensis pair+Metformin	Animal experiments	SD rats	HDL-C↑; BW, FBG, LDL, FPG, PBG, GSP, HOMA-IR↓	Firmicutes, F/B ratio, Lactobacillus, Pediococcus, Clostridium, Dehalobacterium, Ruminococcu↑; Actinobacteria, Bacteroidetes, Butyricicoccus, SMB53, Prevotella↓	T2DM	(Guo et al., 2022)
Metformin			BW, FBG, LDL, FPG, GSP, HOMA-IR↓	Butyricicoccus, SMB53, Clostridium,↑; Lactobacillus, F/B ratio, Prevotella↓		
Xiao-Ke-Yin	Animal experiments	db/db mice	BW, FBG, ITT, insulin, HOMA-IR, ALT, AST, TC, TG, FFA, IL-6, TNF-α↓	Bacilli, Lactobacillaceae and Lactobacillus↑; Clostridia, Lachnospircaeae, Tannerellaceae, Parabacteroides↓	T2DM	(Li M. et al., 2023)
Metformin			BW, FBG, ITT, insulin, HOMA-IR, ALT, AST, TC, TG, FFA, IL-6, TNF-α↓	/		

BW, body weight; FPG, fasting plasma glucose; PBG, Postprandial blood glucose; HbA1c, hemoglobin A1c; TC, total cholesterol; TG, triglyceride; LDL, low-density lipoprotein cholesterol; HDL, high-density lipoprotein cholesterol; HOMA-IR, homeostasis assessment of insulin resistance; FINS, fasting insulin; FFA, free fatty acid; HOMA-ISI, homeostasis assessment of insulin sensitivity index; IL, interleukin; TNF-α, tumor necrosis factor-α; Scr, serum creatinine.

The “↑” symbol is used to indicate an increase, improvement, or positive change in a quantity.

The “↓” symbol is used to indicate a decrease, decline, or negative change.

Our study explores the potential of Traditional Chinese Medicine (TCM) to modulate gut microbiota and influence obesity development. By identifying specific microbiota profiles associated with obesity and using TCM to modulate these microbiota, we can enhance our understanding of obesity pathomechanisms and identify new therapeutic targets. However, these findings warrant validation through clinical trials to confirm the efficacy of TCM in regulating gut flora and the effectiveness of such regulation in obesity treatment. Looking ahead, individualized treatments can be developed based on TCM theories, factoring in the correlations between different TCM physical characteristics and intestinal flora. Furthermore, the holistic concept of TCM allows us to propose a comprehensive obesity control strategy that includes dietary modifications, lifestyle changes, and management of comorbidities. Leveraging the prebiotic-like effects of TCM, new medicinal products can be developed to help modulate IM, promote health, and improve patient compliance. In summary, our findings offer guidance for the development of new herbal formulations, provide clinicians with a rationale for using TCM as a complementary therapy in obesity management, and serve as a practical guide for TCM clinical practice.

### 4.3 Limitations of the study

This study also has its limitations. First, the current version of CiteSpace can only analyze and visualize co-citation graphs for data extracted from SCI-E in the WoSCC database, while data from other databases such as PubMed and Embabe cannot support joint analysis. Second, our search strategy may not include all relevant articles due to search criteria, literature type, and language constraints. In addition, despite the surge in the number of articles published in the field in recent years, the overall number remains relatively small. Therefore, the results of our analysis based on the current literature may be slightly biased. In future studies, we should pay attention to and avoid these limitations as much as possible.

## 5 Conclusion

This research, using CiteSpace, VOSviewer and the Online Analysis Platform of Literature Metrology, provides a comprehensive understanding of the current status, hotspots, and trends in anti-obesity through TCM interventions in IM. China and the United States are leading countries, but countries, institutions, and authors need to collaborate and communicate better. Although research on the modulation of IM by TCM to prevent and treat obesity and its related metabolic diseases has made some progress, there are still some shortcomings. First, most studies are still in the laboratory stage and lack large-scale clinical validation. Second, the quality control and standardization of TCM are also important issues that need to be addressed. Future studies should explore more deeply the mechanism of TCM in regulating IM, as well as the active ingredients and action targets of TCM. At the same time, clinical studies should be strengthened to verify the effectiveness of TCM in regulating intestinal flora to prevent and treat obesity and its related metabolic diseases. In conclusion, this bibliometric study defines the overall prospects and provides valuable information for ongoing studies in this field.

## Data availability statement

The original contributions presented in the study are included in the article/**Supplementary Material**. Further inquiries can be directed to the corresponding authors.

## Author contributions

WH: Data curation, Investigation, Software, Writing – original draft, Visualization. JW: Data curation, Software, Writing – original draft, Visualization. MK: Investigation, Writing – review & editing. ZX: Writing – review & editing, Methodology. BF: Writing – review & editing. GS: Funding acquisition, Supervision, Writing – review & editing. ZT: Supervision, Validation, Writing – review & editing, Methodology.
