# Supplementary material for: Exploring global research status and trends in anti-obesity effects of traditional Chinese medicine through intestinal microbiota: a bibliometric study
Source: Front Cell Infect Microbiol. 2023 Nov 16;13:1271473. doi: 10.3389/fcimb.2023.1271473 (PMC10690589; doi:10.3389/fcimb.2023.1271473)
Supplement: Supplementary file 1 [file Table_1.docx]

Supplementary Material

Exploring global research status and trends in anti-obesity through traditional Chinese medicine interventions in intestinal microbiota: A bibliometric study

Wenjing Huang^1,†^, Jiuyuan Wang^1,†^, Min Kuang^1^, Zixuan Xiao^1^, Boyan Fan^1^, Guixiang Sun^1*^, Zhoujin Tan^1*^

*** Correspondence:** Guixiang Sun [84663423@qq.com;](mailto:84663423@qq.com;) Zhoujin Tan tanzhjin@sohu.com

# Supplementary Tables

**Supplementary Table 1. Top 10 countries annual publications**

|  | **CHINA** | **USA** | **KOREA** | **JAPAN** | **ITALY** | **CANADA** | **SPAIN** | **AUSTRALIA** | **INDIA** | **BELGIUM** |
| --- | --- | --- | --- | --- | --- | --- | --- | --- | --- | --- |
| 2009 | 1 | 0 | 0 | 1 | 0 | 0 | 0 | 0 | 0 | 0 |
| 2010 | 1 | 0 | 0 | 1 | 0 | 0 | 0 | 0 | 0 | 0 |
| 2011 | 1 | 0 | 0 | 1 | 0 | 0 | 0 | 0 | 0 | 0 |
| 2012 | 3 | 0 | 0 | 1 | 0 | 0 | 0 | 0 | 0 | 0 |
| 2013 | 4 | 0 | 0 | 2 | 0 | 0 | 0 | 0 | 0 | 1 |
| 2014 | 5 | 1 | 4 | 2 | 1 | 0 | 0 | 0 | 0 | 1 |
| 2015 | 11 | 4 | 6 | 3 | 1 | 0 | 0 | 0 | 0 | 1 |
| 2016 | 18 | 4 | 8 | 4 | 1 | 1 | 0 | 1 | 0 | 1 |
| 2017 | 34 | 9 | 11 | 5 | 1 | 4 | 1 | 1 | 1 | 2 |
| 2018 | 72 | 17 | 14 | 7 | 2 | 5 | 2 | 5 | 2 | 3 |
| 2019 | 142 | 24 | 17 | 8 | 4 | 6 | 2 | 6 | 3 | 4 |
| 2020 | 226 | 34 | 22 | 19 | 6 | 8 | 4 | 7 | 5 | 5 |
| 2021 | 321 | 37 | 29 | 19 | 8 | 8 | 4 | 9 | 6 | 5 |
| 2022 | 469 | 47 | 37 | 21 | 12 | 9 | 7 | 9 | 8 | 6 |

**Supplementary Table 2. Top 10 foundations**

| **Rank** | **Foundation** | **Count** |
| --- | --- | --- |
| 1 | National Natural Science Foundation Of China | 294 |
| 2 | China Postdoctoral Science Foundation | 21 |
| 3 | National Key Research And Development Program Of China | 35 |
| 4 | Fundamental Research Funds For The Central Universities | 16 |
| 5 | National Natural Science Foundation Of Guangdong Province | 13 |
| 6 | Ministry Of Science And Technology Taiwan | 9 |
| 7 | Ministry Of Science Ict Future Planning Republic Of Korea | 10 |
| 8 | Natural Science Foundation Of Fujian Province | 10 |
| 9 | National Research Foundation Of Korea | 9 |
| 10 | Beijing Natural Science Foundation | 8 |

**Supplementary Table 3. Top 30 authors with the total link strength in the co-occurrence cluster analysis of authors**

| **Rank** | **label** | **x** | **y** | **cluster** | **Weight**  **<Links>** | **Weight**  **<Total link strength>** | **weight** |
| --- | --- | --- | --- | --- | --- | --- | --- |
| 1 | Liu, Bin | -0.3906 | 0.85 | 5 | 2 | 9 | 14 |
| 2 | Kim, Hojun | 0.9989 | 0.1716 | 3 | 3 | 28 | 12 |
| 3 | Bose, Shambhunath | 0.9184 | 0.2658 | 3 | 3 | 28 | 12 |
| 4 | Zhang, Li | -0.9763 | 0.2661 | 7 | 1 | 1 | 12 |
| 5 | Wang, Jing-Hua | 0.9018 | 0.0883 | 3 | 3 | 24 | 9 |
| 6 | Wang, Jing | 0.124 | -0.3314 | 1 | 4 | 4 | 8 |
| 7 | Cani, Patrice D. | -0.8819 | -0.7276 | 6 | 1 | 7 | 7 |
| 8 | Chin, Young-Won | 0.8151 | 0.1869 | 3 | 3 | 20 | 7 |
| 9 | Delzenne, Nathalie M. | -0.8283 | -0.7668 | 6 | 1 | 7 | 7 |
| 10 | He, Xiao-yun | -0.2304 | 0.0911 | 2 | 6 | 32 | 7 |
| 11 | Huang, Kun-lun | -0.3216 | -0.1598 | 2 | 6 | 31 | 7 |
| 12 | Tong, Xiao-lin | -0.0312 | -0.4535 | 1 | 5 | 14 | 7 |
| 13 | Xu, Jia | -0.1339 | -0.2154 | 2 | 10 | 28 | 7 |
| 14 | Zhao, Lin-hua | -0.1143 | -0.5419 | 1 | 3 | 9 | 7 |
| 15 | Li, Hou-kai | 0.7207 | -0.8287 | 8 | 1 | 1 | 6 |
| 16 | Sheng, Yao | -0.2894 | -0.0371 | 2 | 6 | 31 | 6 |
| 17 | Wang, Li | 0.2371 | -0.2863 | 1 | 2 | 4 | 6 |
| 18 | Wang, Qi | 0.3643 | 0.7827 | 4 | 2 | 4 | 6 |
| 19 | Xu, Wen-tao | -0.4264 | -0.0647 | 2 | 6 | 31 | 6 |
| 20 | Yang, Xing-bin | 0.3641 | 0.8522 | 4 | 2 | 4 | 6 |
| 21 | Cui, Huan-tian | 0.2918 | -0.2637 | 1 | 1 | 3 | 5 |
| 22 | Duan, Jin-Ao | -0.9954 | 0.2952 | 7 | 1 | 1 | 5 |
| 23 | Gao, Ze-zheng | -0.019 | -0.5637 | 1 | 3 | 9 | 5 |
| 24 | Li, Bo | 0.7313 | -0.8617 | 8 | 1 | 1 | 5 |
| 25 | Li, Min | 0.0618 | -0.4219 | 1 | 4 | 6 | 5 |
| 26 | Lv, Xu-Cong | -0.3342 | 0.9035 | 5 | 2 | 5 | 5 |
| 27 | Wang, Yu | 0.4243 | 0.8179 | 4 | 2 | 4 | 5 |
| 28 | Zhang, Chuan-hai | -0.3776 | 0.0735 | 2 | 6 | 22 | 5 |
| 29 | Zhao, Chao | -0.442 | 0.907 | 5 | 2 | 6 | 5 |
| 30 | Zheng, Shu-juan | -0.1612 | -0.0276 | 2 | 6 | 26 | 5 |

**Supplementary Table 4. Top 10 organization annual publications**

| **Organization/Year** | **2009** | **2010** | **2011** | **2012** | **2013** | **2014** | **2015** | **2016** | **2017** | **2018** | **2019** | **2020** | **2021** | **2022** | **2023** |
| --- | --- | --- | --- | --- | --- | --- | --- | --- | --- | --- | --- | --- | --- | --- | --- |
| BEIJING UNIVERSITY OF CHINESE MEDICINE | 0 | 0 | 0 | 0 | 0 | 0 | 0 | 0 | 1 | 3 | 2 | 4 | 8 | 14 | 2 |
| CHINESE ACADEMY OF SCIENCES | 1 | 0 | 0 | 2 | 1 | 0 | 0 | 0 | 0 | 2 | 2 | 8 | 9 | 6 | 1 |
| CHINA ACADEMY OF CHINESE MEDICAL SCIENCES | 0 | 0 | 0 | 1 | 0 | 0 | 1 | 0 | 2 | 3 | 4 | 3 | 6 | 10 | 1 |
| SHANGHAI UNIVERSITY OF TRADITIONAL CHINESE MEDICINE | 0 | 0 | 0 | 1 | 0 | 0 | 1 | 1 | 2 | 2 | 5 | 3 | 10 | 3 | 2 |
| NANJING UNIVERSITY OF CHINESE MEDICINE | 0 | 0 | 0 | 0 | 0 | 0 | 1 | 0 | 1 | 1 | 5 | 6 | 4 | 1 | 5 |
| CHENGDU UNIVERSITY OF TRADITIONAL CHINESE MEDICINE | 0 | 0 | 0 | 0 | 0 | 0 | 0 | 0 | 1 | 0 | 2 | 6 | 5 | 5 | 4 |
| SHANGHAI JIAO TONG UNIVERSITY | 0 | 0 | 0 | 1 | 0 | 0 | 1 | 1 | 2 | 2 | 2 | 1 | 3 | 6 | 3 |
| CHINA AGRICULTURAL UNIVERSITY | 0 | 0 | 0 | 0 | 0 | 0 | 0 | 0 | 0 | 1 | 7 | 4 | 1 | 4 | 2 |
| GUANG ANMEN HOSPITAL CACMS | 0 | 0 | 0 | 0 | 0 | 0 | 1 | 0 | 1 | 3 | 1 | 2 | 2 | 6 | 1 |
| ZHEJIANG UNIVERSITY | 0 | 0 | 0 | 0 | 0 | 0 | 0 | 0 | 0 | 0 | 0 | 3 | 4 | 4 | 6 |

**Supplementary Table 5. Co-occurrence cluster analysis of top 20 high-output academic institutions**

| **Rank** | **label** | **x** | **y** | **cluster** | **Weight**  **<Links>** | **Weight**  **<Total link strength>** | **weight** |
| --- | --- | --- | --- | --- | --- | --- | --- |
| 1 | Beijing Univ Chinese Med | 0.098 | 0.8273 | 1 | 7 | 23 | 33 |
| 2 | China Acad Chinese Med Sci | 0.4773 | 0.8335 | 1 | 8 | 29 | 30 |
| 3 | Chinese Acad Sci | 0.2862 | 0.3655 | 5 | 8 | 18 | 30 |
| 4 | Shanghai Univ Tradit Chinese Med | 0.7892 | 0.2653 | 6 | 6 | 13 | 29 |
| 5 | Nanjing Univ Chinese Med | 0.7966 | 0.8323 | 1 | 3 | 4 | 23 |
| 6 | Shanghai Jiao Tong Univ | 0.1881 | 0.2741 | 6 | 7 | 16 | 21 |
| 7 | Chengdu Univ Tradit Chinese Med | 0.2886 | 0.9412 | 1 | 2 | 3 | 20 |
| 8 | China Agr Univ | 0.1354 | 0.6058 | 3 | 2 | 5 | 19 |
| 9 | Zhejiang Univ | 0.8211 | 0.7372 | 2 | 3 | 4 | 17 |
| 10 | China Pharmaceut Univ | 0.6544 | 0.9387 | 1 | 5 | 6 | 16 |
| 11 | Dongguk Univ | 0.462 | 0.2253 | 7 | 0 | 0 | 16 |
| 12 | Zhejiang Chinese Med Univ | 0.587 | 0.6834 | 2 | 4 | 5 | 16 |
| 13 | Guangzhou Univ Chinese Med | 0.1391 | 0.4631 | 4 | 6 | 9 | 14 |
| 14 | Fujian Agr & Forestry Univ | 0.4779 | 0.602 | 3 | 1 | 2 | 13 |
| 15 | Jilin Univ | 0.8303 | 0.6045 | 3 | 1 | 3 | 13 |
| 16 | Tianjin Univ Tradit Chinese Med | 0.3603 | 0.7472 | 2 | 4 | 4 | 13 |
| 17 | Shaanxi Normal Univ | 0.1844 | 0.6959 | 2 | 1 | 1 | 11 |
| 18 | Hunan Agr Univ | 0.6605 | 0.3592 | 5 | 1 | 2 | 10 |
| 19 | Southern Med Univ | 0.4786 | 0.4718 | 4 | 2 | 2 | 10 |
| 20 | Sun Yat Sen Univ | 0.8333 | 0.4693 | 4 | 5 | 5 | 10 |

**Supplementary Table 6. Top 20 highly keywords**

| **Rank** | **Count** | **Centrality** | **Year** | **Keywords** |
| --- | --- | --- | --- | --- |
| 1 | 477 | 0.05 | 2012 | gut microbiota |
| 2 | 194 | 0.02 | 2013 | obesity |
| 3 | 176 | 0.07 | 2012 | high-fat diet |
| 4 | 154 | 0.09 | 2012 | inflammation |
| 5 | 144 | 0.06 | 2012 | insulin-resistance |
| 6 | 86 | 0.07 | 2012 | extract |
| 7 | 78 | 0.04 | 2012 | metabolism |
| 8 | 77 | 0.02 | 2015 | mice |
| 9 | 75 | 0.07 | 2017 | short-chain fatty acids |
| 10 | 56 | 0.02 | 2018 | type 2 diabetes mellitus |
| 11 | 55 | 0.05 | 2016 | akkermansia-muciniphila |
| 12 | 55 | 0.1 | 2012 | acid |
| 13 | 52 | 0.02 | 2018 | lipid-metabolism |
| 14 | 52 | 0.02 | 2012 | mechanisms |
| 15 | 52 | 0.02 | 2017 | health |
| 16 | 50 | 0.03 | 2018 | non-alcoholic fatty liver disease |
| 17 | 49 | 0.04 | 2014 | diet |
| 18 | 47 | 0.07 | 2017 | oxidative stress |
| 19 | 47 | 0.02 | 2019 | traditional chinese medicine |
| 20 | 43 | 0.02 | 2018 | metabolic syndrome |

**Supplementary Table 7. Summary of the largest 11 clusters**

| **ClusterID** | **Size** | **Label (LSI)** | **Label (LLR)** | **Label (MI)** | **Average Year** |
| --- | --- | --- | --- | --- | --- |
| 0 | 95 | gut microbiota | traditional chinese medicine (295.02, 1.0E-4) | duyun compound (2.45) | 2016 |
| 1 | 63 | gut microbiota | gut microbiota composition (205.91, 1.0E-4) | modulation effect (0.96) | 2017 |
| 2 | 61 | gut microbiota | akkermansia muciniphila (285.95, 1.0E-4) | chinese herbal product (1.27) | 2015 |
| 3 | 58 | gut microbiota | herbal medicine (184.64, 1.0E-4) | diet-induced non-alcoholic fatty liver disease (0.79) | 2019 |
| 4 | 49 | gut microbiota | obese middle-aged korean women (71.56, 1.0E-4) | gut microbiota (0.09) | 2012 |
| 5 | 49 | gut microbiota | gut permeability (100.36, 1.0E-4) | duyun compound (0.1) | 2013 |
| 6 | 46 | gut microbiota | metabolic disorder (190, 1.0E-4) | duyun compound (0.75) | 2018 |
| 7 | 44 | gut microbiota | oral bioavailability conundrum (97.94, 1.0E-4) | gut microbiota (0.07) | 2013 |
| 8 | 40 | gut microbiota | yinchen linggui zhugan decoction (112.31, 1.0E-4) | enterococcus cecorum abundance (0.65) | 2018 |
| 9 | 14 | curcuma longa | subcutaneous adipose tissue (25.26, 1.0E-4) | gut microbiota (0.11) | 2010 |
| 11 | 8 | gut microbiota modulation | gut microbiota modulation (75.85, 1.0E-4) | gut microbiota (0.1) | 2014 |

**Supplementary Table 8. Top 20 highly cited articles**

| **Rank** | **Count** | **Centrality** | **Year** | **CITED REF** |
| --- | --- | --- | --- | --- |
| 1 | 48 | 0.07 | 2015 | Chang CJ, 2015, NAT COMMUN, V6, P0, DOI 10.1038/ncomms8489 |
| 2 | 41 | 0.05 | 2019 | Wu TR, 2019, GUT, V68, P248, DOI 10.1136/gutjnl-2017-315458 |
| 3 | 38 | 0.06 | 2018 | Zhao LP, 2018, SCIENCE, V359, P1151, DOI 10.1126/science.aao5774 |
| 4 | 30 | 0.14 | 2015 | Anhe FF, 2015, GUT, V64, P872, DOI 10.1136/gutjnl-2014-307142 |
| 5 | 30 | 0.08 | 2015 | Xu J, 2015, ISME J, V9, P552, DOI 10.1038/ismej.2014.177 |
| 6 | 28 | 0.01 | 2019 | Canfora EE, 2019, NAT REV ENDOCRINOL, V15, P261, DOI 10.1038/s41574-019-0156-z |
| 7 | 27 | 0.04 | 2018 | Tong XL, 2018, MBIO, V9, P0, DOI 10.1128/mBio.02392-17 |
| 8 | 26 | 0.05 | 2018 | Wei XY, 2018, SCI REP-UK, V8, P0, DOI 10.1038/s41598-018-22094-2 |
| 9 | 24 | 0.05 | 2016 | Koh A, 2016, CELL, V165, P1332, DOI 10.1016/j.cell.2016.05.041 |
| 10 | 23 | 0.06 | 2018 | Chen GJ, 2018, MOL NUTR FOOD RES, V62, P0, DOI 10.1002/mnfr.201700485 |
| 11 | 22 | 0.06 | 2013 | Everard A, 2013, P NATL ACAD SCI USA, V110, P9066, DOI 10.1073/pnas.1219451110 |
| 12 | 21 | 0.07 | 2017 | Xu J, 2017, MED RES REV, V37, P1140, DOI 10.1002/med.21431 |
| 13 | 21 | 0.01 | 2014 | Shin NR, 2014, GUT, V63, P727, DOI 10.1136/gutjnl-2012-303839 |
| 14 | 20 | 0.07 | 2016 | Dao MC, 2016, GUT, V65, P426, DOI 10.1136/gutjnl-2014-308778 |
| 15 | 19 | 0.04 | 2019 | Huang FJ, 2019, NAT COMMUN, V10, P0, DOI 10.1038/s41467-019-12896-x |
| 16 | 19 | 0.04 | 2017 | Yan HL, 2017, PHYTOMEDICINE, V26, P45, DOI 10.1016/j.phymed.2017.01.007 |
| 17 | 19 | 0.02 | 2017 | Zhao L, 2017, FOOD FUNCT, V8, P4644, DOI 10.1039/C7FO01383C |
| 18 | 19 | 0.01 | 2019 | Wang K, 2019, CELL REP, V26, P222, DOI 10.1016/j.celrep.2018.12.028 |
| 19 | 18 | 0.03 | 2015 | Roopchand DE, 2015, DIABETES, V64, P2847, DOI 10.2337/db14-1916 |
| 20 | 18 | 0.01 | 2015 | Canfora EE, 2015, NAT REV ENDOCRINOL, V11, P577, DOI 10.1038/nrendo.2015.128 |
| 21 | 18 | 0.01 | 2015 | Zhang XQ, 2015, SCI REP-UK, V5, P0, DOI 10.1038/srep10737 |

**Supplementary Table 9. Top 10 highly centrality articles**

| **Rank** | **Centrality** | **References** | **DOI** | **Cluster ID** |
| --- | --- | --- | --- | --- |
| 1 | 0.13 | Anhe FF, 2015, GUT, V64, P872 | 10.1136/gutjnl-2014-307142 | 2 |
| 2 | 0.13 | Martel J, 2017, NAT REV ENDOCRINOL, V13, P149 | 10.1038/nrendo.2016.142 | 0 |
| 3 | 0.11 | Cao Y, 2016, ARCH IRAN MED, V19, P197 |  | 2 |
| 4 | 0.09 | Xu J, 2015, ISME J, V9, P552 | 10.1038/ismej.2014.177 | 0 |
| 5 | 0.09 | Amaretti A, 2015, NUTRIENTS, V7, P2788 | 10.3390/nu7042788 | 7 |
| 6 | 0.08 | Xu J, 2017, MED RES REV, V37, P1140 | 10.1002/med.21431 | 0 |
| 7 | 0.08 | Everard A, 2014, ISME J, V8, P2116 | 10.1038/ismej.2014.45 | 4 |
| 8 | 0.08 | Dao MC, 2016, GUT, V65, P426 | 10.1136/gutjnl-2014-308778 | 2 |
| 9 | 0.08 | Ridaura VK, 2013, SCIENCE, V341, P1079 | 10.1126/science.1241214 | 4 |
| 10 | 0.08 | Chang CJ, 2015, NAT COMMUN, V6, P0 | 10.1038/ncomms8489 | 0 |
